# Photonic Doping of Epsilon‐Near‐Zero Bragg Microcavities

**DOI:** 10.1002/nap2.70186

**Published:** 2026-06-26

**Authors:** Ali Panahpour, Jussi Kelavuori, Mikko Huttunen

**Affiliations:** ^1^ Photonics Laboratory, Physics Unit Tampere University Tampere Finland

**Keywords:** epsilon‐near‐zero photonics, magnetic purcell factor, Mie resonators, photonic doping

## Abstract

Epsilon‐near‐zero (ENZ) photonics provides a powerful route to extreme dispersion engineering, strong field confinement, and unconventional wave phenomena. A closely related concept, *photonic doping*, employs subwavelength nonmagnetic dielectric inclusions in ENZ media to enable exotic responses such as perfect‐magnetic‐conductor behavior and simultaneous epsilon‐and mu‐near‐zero states. However, photonic doping has remained limited to microwave and far‐infrared regimes due to intrinsic losses in optical ENZ materials. Here, photonic doping is demonstrated at optical frequencies by embedding a periodic array of dielectric Mie resonators within an ultralow‐loss all‐dielectric ENZ platform based on near‐cutoff Bragg microcavities. The resulting structures support spectrally isolated quasi‐singular coupled Bragg–Mie resonances spanning electric and magnetic multipolar orders and their overtones. These modes exhibit effective near‐zero‐index dispersion, with fields confined either within or between the nanoparticles. A representative 18μm‐scale doped structure exhibits a quality factor of $3.4\times 10^{4}$ and a magnetic Purcell enhancement exceeding $1.8\times 10^{4}$ in the near‐infrared. This platform elevates photonic doping from a microwave‐only concept to a fully optical, low‐loss, and multipole‐resolved system, enabling ultra‐narrowband Mie‐like resonances, enhanced magnetic–light interactions, and new opportunities in multipolar‐selective spectroscopy and lasing, low‐threshold nonlinear optics, and engineered spontaneous emission.

## Graphical abstract

Photonic doping of an epsilon‐near‐zero (ENZ) Bragg microcavity at optical frequencies induces strong magnetic responses from dielectric inclusions, enabling intense field localization, Purcell enhancement, and tunable hybrid Bragg–Mie modes. The platform supports perfect magnetic conductor and epsilon‐and‐mu‐near‐zero regimes, offering opportunities for enhanced light–matter interaction, multipolar spectroscopy, low‐threshold nonlinear optics, and controlled emission.

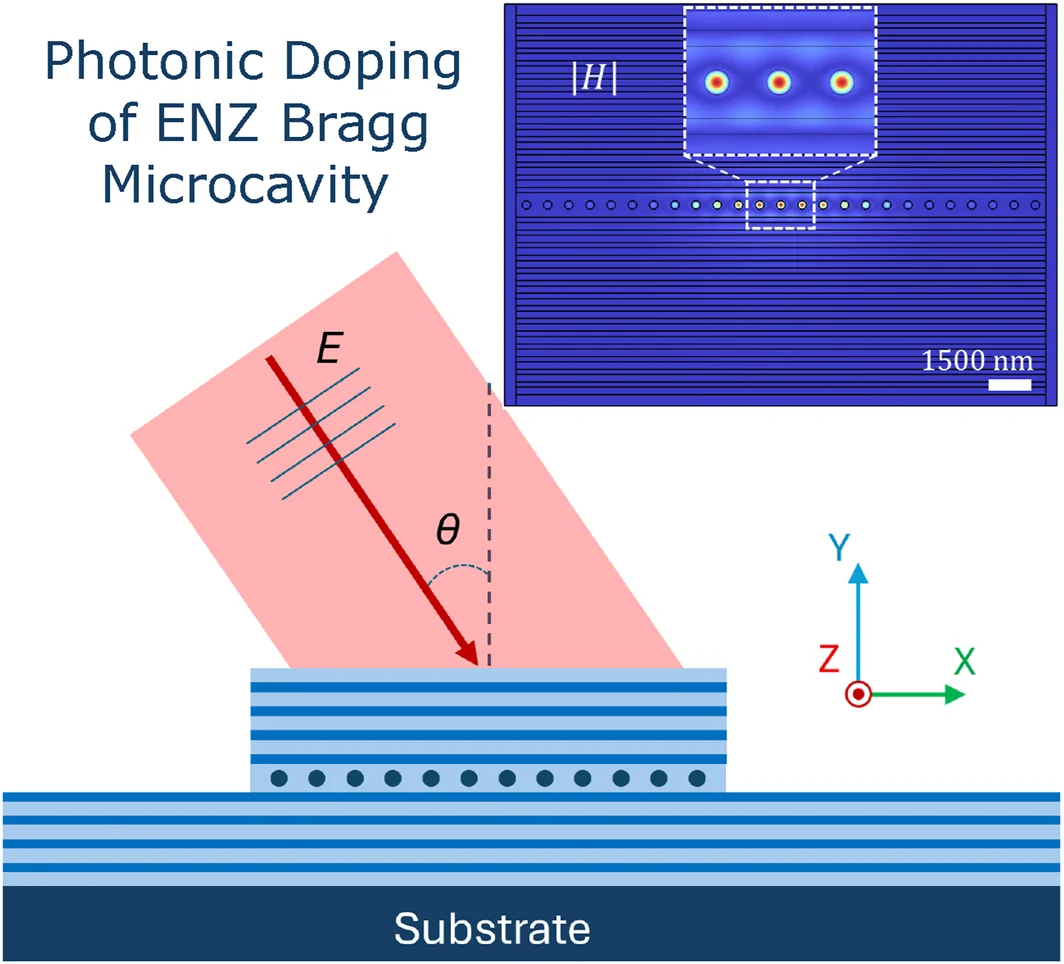

## 1 Introduction

Controlling light at the nanoscale is a central objective of nanophotonics, fundamentally governed by the tailoring of light–matter interactions via the material parameters permittivity (ε) and permeability (μ), which together determine the refractive index n. Advances in materials and nanofabrication have enabled precise control over these parameters, giving rise to a wide range of engineered optical responses. Among these, near‐zero‐index (NZI) photonics has emerged as a particularly intriguing regime, encompassing materials in which the refractive index approaches zero due to vanishing ε, μ, or both, referred to as epsilon‐near‐zero (ENZ), mu‐near‐zero (MNZ), and epsilon‐and‐mu‐near‐zero (EMNZ) media [1, 2]. In such systems, the effective wavelength diverges, the phase velocity becomes unbounded, and the wavevector approaches zero. However, different NZI classes exhibit markedly different group velocities, impedances, and field‐confinement properties [3], such that the physical origin of the NZI response governs the unconventional functionalities they support, including supercoupling, diffraction suppression, cloaking, and enhanced light–matter interactions [1].

A powerful approach to functionalize ENZ media is through *photonic doping* [4, 5, 6], whereby subwavelength dielectric inclusions modify or encode new electromagnetic properties, analogously to electronic doping in semiconductors. This concept has enabled extraordinary effects such as tunneling through ENZ media or general impedance matching [7, 8, 9, 10], geometry‐independent radiation of antennas [11], dispersion coding [12], enhanced magneto‐optical [13] and Kerr [14] effects, ultra‐sensitive switching and displacement sensing [15], strong concentration of magnetic fields [16], and the realization of perfect magnetic conductor (PMC) and EMNZ behavior using non‐magnetic components [6, 7]. However, material losses and fabrication challenges at optical wavelengths have so far limited the photonic doping of ENZ media primarily to microwave [6, 9, 10, 14] and far‐infrared frequencies^1^ [18].

Recently, ENZ Bragg microcavities operating near cutoff have been proposed as an alternative all‐dielectric route to optical ENZ behavior [19, 20]. In these structures, the ENZ response arises from a near‐cutoff waveguide mode supported within a Bragg‐reflection microcavity, providing an optical ENZ host without relying on lossy ENZ materials and plasmonic structures [21, 22]. Embedding dielectric Mie resonators into such a host creates a new regime of *optical photonic doping* in which the resonant response of the inclusions is strongly reshaped by the ENZ environment. In contrast to isolated dielectric nanoparticles (NPs), whose Mie resonances are intrinsically radiative and therefore limited in quality (Q) factor, dopants embedded in an ENZ Bragg cavity experience strong suppression of radiative leakage. This leads to spectrally sharp and spatially confined hybrid Bragg–Mie resonances with strongly enhanced electric or magnetic local density of states (LDOS). Such predominantly single‐multipole, spectrally isolated modes are especially attractive for selective Purcell enhancement, magnetic‐dipole spectroscopy, multipolar‐selective lasing, and controlled studies of higher‐order optical transitions in atoms, molecules, or quantum emitters.

This platform is also conceptually distinct from other high‐Q dielectric photonic systems. In metasurfaces and photonic‐crystal slabs, the suppression of radiation commonly arises from collective interference effects, symmetry protection, or bound states in the continuum (BICs), where the modal properties are dictated by lattice‐mediated coupling and Bloch‐mode structure [23, 24, 25, 26, 27]. By contrast, in photonically doped ENZ Bragg cavities, radiative suppression is already provided by the ENZ cavity background itself, whereas the dielectric inclusions introduce electric or magnetic Mie resonances that hybridize with the ENZ modes. The resulting hybrid modes therefore combine ENZ‐mediated radiation suppression with dopant‐controlled multipolar response. This offers a route to high‐Q and highly selective resonances that does not rely primarily on collective lattice cancellation but instead on the interplay between an ENZ host and resonant dielectric dopants.

Moreover, the photonic doping of Bragg microcavities introduces a fundamentally distinct mechanism for realizing optical EMNZ behavior. Conventional all‐dielectric zero‐index platforms [28, 29, 30, 31, 32] typically rely on photonic–crystal band engineering, such as Dirac‐cone or accidental‐degeneracy physics, in which effective ε and μ emerge simultaneously from Bloch‐mode dispersion near a degeneracy point and are therefore intrinsic to the band structure. In contrast, the EMNZ/PMC response in the optical photonic‐doping platform originates from the interplay of two physically distinct subsystems: the ENZ host and the magnetic Mie resonance of the embedded inclusions which gives rise to the magnetic permeability pole. The resulting EMNZ response is therefore host–dopant driven rather than band‐degeneracy driven, making it conceptually much closer to the original photonic‐doping paradigm than to Dirac‐cone zero‐index systems.

Another important characteristic of this platform is that the resulting EMNZ regime is strongly dispersive. Unlike Dirac‐cone zero‐index systems, which exhibit nearly linear dispersion in the vicinity of the degeneracy point, the hybrid Bragg–Mie platform displays rapid spectral variation of the effective refractive index within the NZI region. This steep dispersion implies a large group index and slow‐light behavior, which can lead to enhanced Q‐factor and LDOSs, as radiative processes depend sensitively on the spectral variation of the electromagnetic response [3].

The improved Q‐factor and magnetic Purcell enhancement provided by this platform are directly relevant to magnetic light–matter interactions [33, 34, 35, 36] and to nonlinear nanophotonics, where enhanced magnetic fields can boost Kerr effects [14], second‐ and third‐harmonic generation [37, 38], and Raman scattering [39, 40]. More broadly, the platform provides a route to spectrally isolated high‐Q predominantly single‐multipole resonances in an all‐dielectric optical system, which is difficult to achieve using existing beam‐engineering [41, 42], self‐interference [43, 44, 45], plasmonic [46, 47, 48], or anapole‐based strategies [33, 49, 50].

These distinctive features of the proposed photonically doped ENZ Bragg microcavity define the central contribution of this study. By embedding resonant dielectric inclusions into an all‐dielectric ENZ Bragg microcavity, we extend the concept of photonic doping of ENZ media from its established microwave and far‐infrared implementations to optical frequencies. In this platform, the Bragg microcavity does not merely provide an alternative geometry but acts as a low‐loss optical ENZ host that suppresses radiative leakage and enables high‐Q hybrid Bragg–Mie resonances from embedded dielectric dopants. The resulting effective EMNZ and PMC‐like responses arise from a host–dopant‐driven mechanism, in which the ENZ response is supplied by the Bragg microcavity while the embedded nonmagnetic dielectric inclusions provide resonant electric or magnetic polarizability. This mechanism is physically distinct from Dirac‐cone, accidental‐degeneracy, BIC, or lattice‐interference‐based zero‐index and high‐Q platforms, and provides a route to engineered zero‐index responses and magnetic light–matter interactions at optical frequencies.

Here, we investigate hybrid Bragg–Mie modes arising from dielectric inclusions embedded in all‐dielectric ENZ Bragg microcavities. We consider both spherical and cylindrical inclusions in ENZ backgrounds, with particular emphasis on periodic arrays of dielectric nanocylinders (NCs) embedded within ENZ Bragg cavities; the implementation of rectangular‐cross‐section inclusions is discussed in Supporting Information S1: Section G. We show that the proposed host–dopant system supports optical EMNZ behavior and NZI Bragg–Mie modes with substantially enhanced Q‐factors and Purcell factors on the order of $10^{4}$, significantly exceeding those of both undoped cavities and isolated NC arrays. Section 2 presents analytical results for Mie resonances in ENZ media, Section 3 provides numerical analysis of photonic doping in ENZ Bragg cavities, and Section 4 discusses fabrication feasibility, robustness against optical losses, and future opportunities enabled by this approach.

## 2 Analytical Study of Mie Resonances in ENZ Media

In the following theoretical and numerical analysis of the influence of ENZ media on Mie resonances, we assume an effectively lossless—a high figure‐of‐merit (FoM$=\varepsilon_{r}/\varepsilon_{i}$)—regime defined by $\varepsilon_{i}\ll \varepsilon_{r}\ll 1$, where $\varepsilon =\varepsilon_{r}+i\varepsilon_{i}$ denotes the complex relative permittivity. In this limit, the FoM also closely approximates the ratio of the real to imaginary components of the complex refractive index, given by $FoM=\left(n^{2}-\kappa^{2}\right)/\left(2n\kappa \right)\approx n/2\kappa$, because $\varepsilon_{i}\ll \varepsilon_{r}\ll 1$ implies κ≪n≪1. This assumption is motivated by the fact that the Q‐factor of Mie resonances can be significantly enhanced only under such low‐loss ENZ conditions [19].

In this section, we provide an analytical demonstration of this effect for spherical and cylindrical dielectric objects. We begin by applying the Mie theory to an infinitely long dielectric cylinder illuminated by a normally incident plane wave with wavenumber k. The field polarization convention and Mie coefficient notation follow the standard formulation of cylindrical scattering theory [51, 52] (see Supporting Information S1: Section A). When the electric field is polarized perpendicular to the cylinder axis (TE polarization), the extinction efficiency $Q_{\text{ext}}$ is proportional to the real part of the scattering coefficients $a_{n}$ [52], given by the following equation:

(1) $$a_{n}=\frac{x_{i}J_{n}\left(x_{i}\right)J_{n}^{\prime }\left(x_{e}\right)-x_{e}J_{n}\left(x_{e}\right)J_{n}^{\prime }\left(x_{i}\right)}{x_{i}J_{n}\left(x_{i}\right){H_{n}^{\left(1\right)}}^{\prime }\left(x_{e}\right)-x_{e}H_{n}^{\left(1\right)}\left(x_{e}\right)J_{n}^{\prime }\left(x_{i}\right)},$$

where $J_{n}$ and $H_{n}^{\left(1\right)}$ denote the Bessel and Hankel functions of the first kind, respectively, n is an integer index labeling the angular (multipolar) order of the cylindrical harmonics and $J_{n}^{\prime }$ corresponds to the derivative of $J_{n}$. The size parameters are defined as $x_{i}=n_{i}kR$ and $x_{e}=n_{e}kR$, corresponding to the arguments of the Bessel and Hankel functions inside and outside the cylinder. In these expressions, $n_{i}$ and $n_{e}$ denote the refractive indices of the cylinder and the surrounding medium, k is the vacuum wavenumber, and R is the radius of the cylinder.

In Figure 1a, the real parts of the Mie coefficients $a_{0}$, $a_{1}$, and $a_{2}$ are plotted as the functions of wavelength, representing the electric and magnetic resonances of an infinite dielectric cylinder with radius R=150nm. The cylinder $\left(n_{i}=3.5\right)$ is embedded in a medium with $n_{e}=1$ and is illuminated by a normally incident plane wave whose electric field is perpendicular to the cylinder axis (see inset).

**FIGURE 1:**
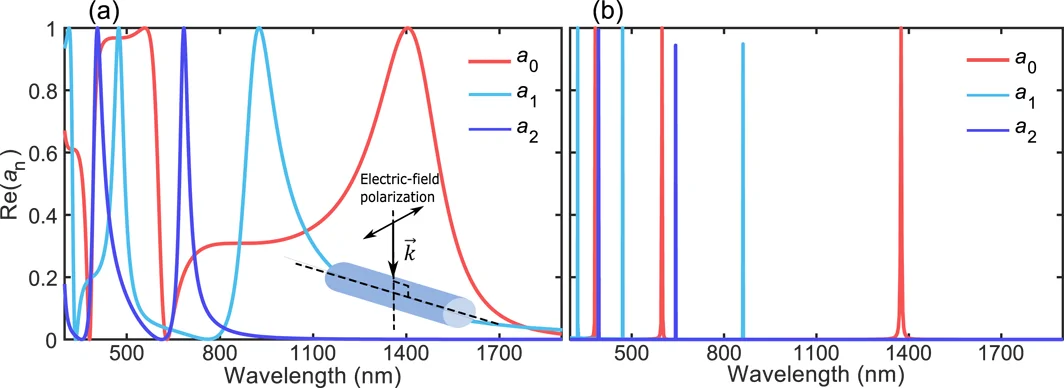
Real parts of Mie coefficients $a_{0}$, $a_{1}$, and $a_{2}$ as functions of wavelength for an infinite cylinder of radius R=150nm, $n_{i}=3.5$ and (a) $n_{e}=1$, (b) $n_{e}=0.1$. The inset illustrates normal incidence of a plane wave with its electric field polarized perpendicular to the cylinder axis.

Figure 1b presents the same Mie coefficients after adjusting the refractive index of the surrounding medium to $n_{e}=0.1$, which results in a pronounced narrowing of the resonances. In particular, for the ${\text{TE}}_{0}$ magnetic dipole mode (corresponding to coefficient $a_{0}$), the bandwidth decreases from approximately 300 nm down to around 2nm. We note that a dispersionless and purely real index $n_{i}=3.5$ has been used over the entire spectral range of λ=300–1900nm to decouple geometry‐driven modal physics from line‐shaping effects due to dispersion. In this idealized setting, clear bandwidth narrowing takes place for all electric and magnetic modes in the ENZ regime. Material dispersion and loss would mainly add spectral shifts and broadening, masking but not altering the underlying mechanism.

This resonance narrowing in the ENZ regime, where $n_{e}\to 0$ and $x_{e}\ll 1$, can be analytically justified by applying Equation (1) and using the small‐argument approximations for the functions $J_{n}\left(x_{e}\right)$, $J_{n}^{\prime }\left(x_{e}\right)$, $H_{n}^{\left(1\right)}\left(x_{e}\right)$, and ${H_{n}^{\left(1\right)}}^{\prime }\left(x_{e}\right)$. As demonstrated in Supporting Information S1: Section A, this procedure yields the resonance condition $J_{n}\left(x_{i}\right)=0$ (consistent with the results shown in Figure 1b), whereas for the resonance width $\left(\gamma_{n}\right)$ of the ${\text{TE}}_{n}$ modes, we obtain the following equation:

(2) $$\gamma_{n}\propto \frac{1}{2\left(n+1\right)!}\left[x_{e}^{2}J_{n}^{\prime }\left(x_{i}\right)\right]{\left(\frac{x_{e}}{2}\right)}^{n+1},$$

which vanishes in the limit $x_{e}\to 0$.

For the case of TM modes, where the electric field is polarized along the cylinder axis (shown in the inset of Figure 2a), the extinction efficiency is proportional to the real part of the scattering coefficients $b_{n}$, expressed as follows:

(3) $$b_{n}=\frac{x_{e}J_{n}\left(x_{i}\right)J_{n}^{\prime }\left(x_{e}\right)-x_{i}J_{n}\left(x_{e}\right)J_{n}^{\prime }\left(x_{i}\right)}{x_{e}J_{n}\left(x_{i}\right){H_{n}^{\left(1\right)}}^{\prime }\left(x_{e}\right)-x_{i}H_{n}^{\left(1\right)}\left(x_{e}\right)J_{n}^{\prime }\left(x_{i}\right)}.$$

**FIGURE 2:**
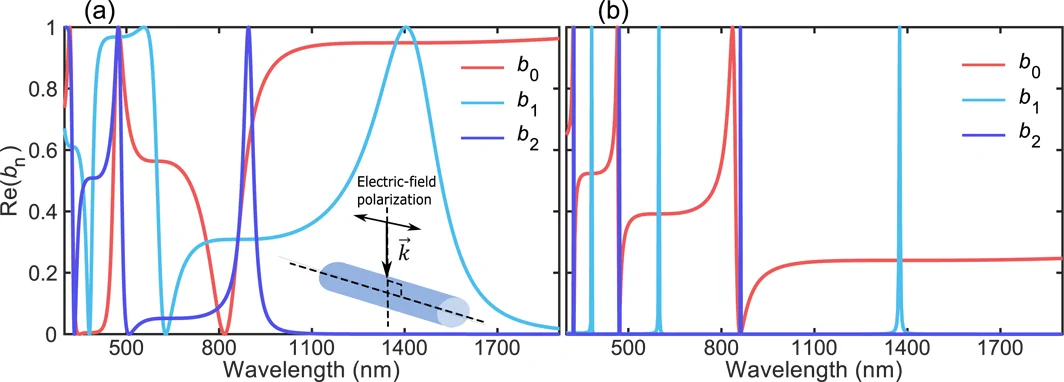
Real parts of Mie coefficients $b_{0}$, $b_{1}$, and $b_{2}$ as functions of wavelength using the same parameters as in Figure 1 and for (a) $n_{e}=1$, (b) $n_{e}=0.1$.

The real parts of the Mie coefficients $b_{0}$, $b_{1}$, and $b_{2}$ are plotted in Figure 2. Again, significant narrowing of resonances takes place when moving from $n_{e}=1$ to $n_{e}=0.1$. By using similar small‐argument approximations for Bessel and Hankel functions, it can be shown (Supporting Information S1: Section A) that the resonance condition for the ${\text{TM}}_{n}$ modes with n>0 is satisfied when it is met as follows:

(4) $$\frac{n}{2}J_{n}\left(x_{i}\right)+x_{i}J_{n}^{\prime }\left(x_{i}\right)=0.$$

However, as discussed in Supporting Information S1: Section A, the ${\text{TM}}_{0}$ mode which corresponds to a cylindrically symmetric electric monopole field distribution does not support a true resonance as can also be inferred from the red curve in Figure 2b. For the resonance width of the ${\text{TM}}_{n}$ modes with n>0 we get the following equation:

(5) $$\gamma_{n}\propto \frac{1}{n!}\left[\frac{n}{2}J_{n}\left(x_{i}\right)-x_{i}J_{n}^{\prime }\left(x_{i}\right)\right]{\left(\frac{x_{e}}{2}\right)}^{n},$$

which vanishes in the ENZ regime as $x_{e}\to 0$, indicating strong suppression of radiative losses and enhanced confinement.

The Q‐factor enhancement and slight blue shift of Mie resonances in the ENZ regime, as demonstrated in Figures 1 and 2, originate from the NZI background surrounding the dielectric NC. In this regime, the vanishing wavevector in the ENZ medium suppresses radiative leakage and strongly inhibits field penetration into the surrounding region. As a result, the Mie resonances become more spatially confined and spectrally narrower, leading to enhanced Q‐factors. The increased confinement also reduces the effective mode volume, causing the particle to behave as a smaller optical cavity and shifting the resonance to shorter wavelengths [53].

Similar effects can be observed for a spherical particle of radius R, characterized by the size parameters $x_{i}=n_{i}kR$ and $x_{e}=n_{e}kR$, corresponding to the interior and exterior of the sphere, respectively. When the sphere is illuminated with a plane wave with wavenumber k, the Mie coefficients for the electric $\left(a_{n}\right)$ and magnetic $\left(b_{n}\right)$ multipole modes can be written in terms of the spherical Bessel functions $j_{n}\left(x\right)$ and $h_{n}^{\left(1\right)}\left(x\right)$ or equivalently the Riccati–Bessel functions $\psi_{n}\left(x\right)=xj_{n}\left(x\right)$ and $\xi_{n}\left(x\right)=xh_{n}^{\left(1\right)}\left(x\right)$ as follows:

(6) $$a_{n}=\frac{x_{i}\psi_{n}\left(x_{i}\right)\psi_{n}^{\prime }\left(x_{e}\right)-x_{e}\psi_{n}\left(x_{e}\right)\psi_{n}^{\prime }\left(x_{i}\right)}{x_{i}\psi_{n}\left(x_{i}\right)\xi_{n}^{\prime }\left(x_{e}\right)-x_{e}\xi_{n}\left(x_{e}\right)\psi_{n}^{\prime }\left(x_{i}\right)},$$

(7) $$b_{n}=\frac{x_{e}\psi_{n}\left(x_{i}\right)\psi_{n}^{\prime }\left(x_{e}\right)-x_{i}\psi_{n}\left(x_{e}\right)\psi_{n}^{\prime }\left(x_{i}\right)}{x_{e}\psi_{n}\left(x_{i}\right)\xi_{n}^{\prime }\left(x_{e}\right)-x_{i}\xi_{n}\left(x_{e}\right)\psi_{n}^{\prime }\left(x_{i}\right)}.$$

In Supporting Information S1: Section B, it is shown analytically that in the ENZ regime $\left(x_{e}\to 0\right)$, the Q‐factors of all electric and magnetic dipolar and higher‐order resonances are enhanced. To illustrate this effect, the real parts of Mie coefficients, corresponding to electric dipole $\left(a_{1}\right)$, electric quadrupole $\left(a_{2}\right)$, magnetic dipole $\left(b_{1}\right)$, and magnetic quadrupole $\left(b_{2}\right)$ resonances of a spherical NP of radius R=85nm and refractive index $n_{i}=3.4$ are plotted for two cases of $n_{e}=1$ and $n_{e}=0.1$, shown in Figure 3a,b, respectively.

**FIGURE 3:**
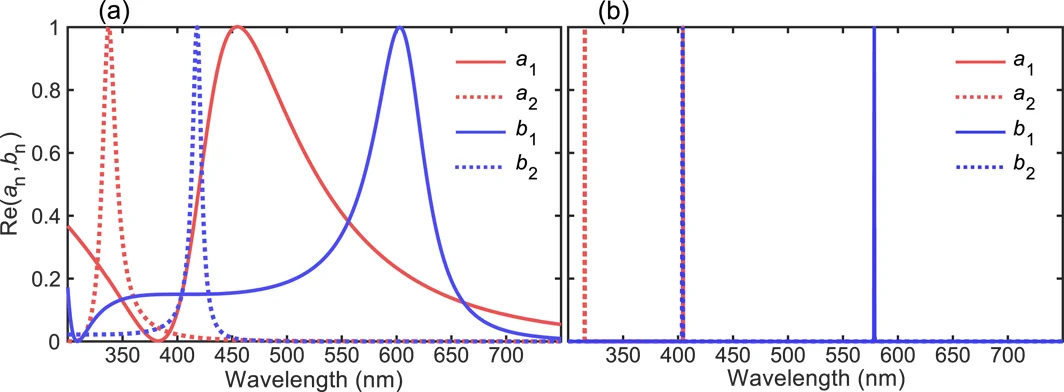
Real parts of Mie coefficients as functions of wavelength for a spherical NP of radius R=85 nm and refractive index $n_{i}=3.4$, inside a medium with refractive index of (a) $n_{e}=1$, (b) $n_{e}=0.1$.

These results extend the analysis of Ref. [54] on Mie resonances of spherical particles in the zero‐epsilon limit. Although that work predicts the suppression of electric resonances as ε→0, our analysis demonstrates that electric resonances persist and evolve into high‐Q modes as the surrounding permittivity approaches zero. This discrepancy originates from the asymptotic treatment employed in Ref. [54], which does not capture the narrow resonance regime, and highlights the need for a more general analysis, as provided in SI, Sec. B.

## 3 Numerical Study of Photonic Doping in ENZ Bragg Cavities

As outlined in the Introduction, most demonstrations of photonic doping in ENZ media have been carried out at microwave frequencies, typically using near‐cutoff metallic or virtual PEC waveguides to emulate ENZ behavior. In such structures, even a single dielectric inclusion can strongly modify the effective electromagnetic response, giving rise to PMC‐ or EMNZ‐like behavior [6]. By contrast, analogous effects do not clearly emerge when a single NP is embedded in the core of an ENZ Bragg cavity. In this geometry, a periodic array of NPs is required to observe the photonic‐doping features. This difference originates from the distinct boundary conditions imposed by PEC and Bragg mirrors. In a near‐cutoff metallic waveguide, the metallic boundaries confine the electromagnetic field to essentially a single ENZ‐guided mode, with no open radiation continuum. The structure is effectively a closed system, and a dielectric inclusion predominantly couples to and perturbs this single confined mode. As a result, even a single defect, regardless of its position can strongly perturb the near‐cutoff mode, producing a sharp Mie resonance and the associated effective PMC or EMNZ response.

In contrast, an ENZ Bragg microcavity is an open resonant system that supports not only the desired near‐cutoff core‐confined mode but also a continuum of leaky and weakly confined radiation channels. As a result, a single dielectric inclusion introduced into such a cavity couples not only to the near‐cutoff mode responsible for the ENZ response but also to these radiation channels, leading to increased radiative loss and a reduced effective Q‐factor. To suppress this radiative coupling, we employ a periodic array of inclusions in the Bragg cavity structure. The periodicity enforces Bloch symmetry, restricting the electromagnetic response to discrete in‐plane momentum components. Since only harmonics within the external light cone correspond to radiative channels, this momentum selectivity suppresses coupling to undesired leakage pathways. At the same time, it ensures that only those components phase‐matched to the near‐cutoff cavity mode contribute significantly, thereby enabling efficient hybridization between the ENZ mode and the dopant Mie resonances. As a result, the electromagnetic response becomes concentrated in well‐defined Bragg–Mie hybrid modes with reduced radiative loss. This collective loading mechanism enables the emergence of sharp resonances and the associated PMC/EMNZ features in the Bragg‐cavity platform.

In the dense‐array limit, strong inter‐particle coupling and lattice‐induced collective effects modify the Bloch‐mode structure [55] and increase radiative leakage, resulting in broader resonances and reduced Q‐factors even in the absence of material loss. In addition, strong near‐field variations across the closely spaced inclusions in this regime can lead to mixing of higher‐order Mie contributions, providing additional channels for radiative damping. As the lattice period is increased, inter‐particle coupling weakens whereas the array still supports a well‐defined Bloch mode compatible with Bragg confinement. In this intermediate regime, as shown in this section, the coupled Bragg–Mie resonance red‐shifts by increasing the period and the Q‐factor increases, as radiative leakage is minimized while strong field localization inside the NPs is maintained. When the period becomes too large, however, the NPs no longer act as an effective collective photonic dopant; the modal weight inside each NP becomes small and the dominant resonances shift to modes with fields mostly concentrated between the particles or within the Bragg stacks. The hybrid Bragg–Mie mode then disappears and is replaced by other cavity resonances with weak NP participation.

To numerically investigate these effects and the photonic doping features of ENZ cavities, we examine the resonance behavior of a periodic array of infinitely long dielectric cylinders embedded in ENZ Bragg cavities. Two‐dimensional COMSOL simulations are performed to calculate the transmittance of the structure shown in Figure 4, and the eigenmodes are obtained using the mode analysis module.

**FIGURE 4:**
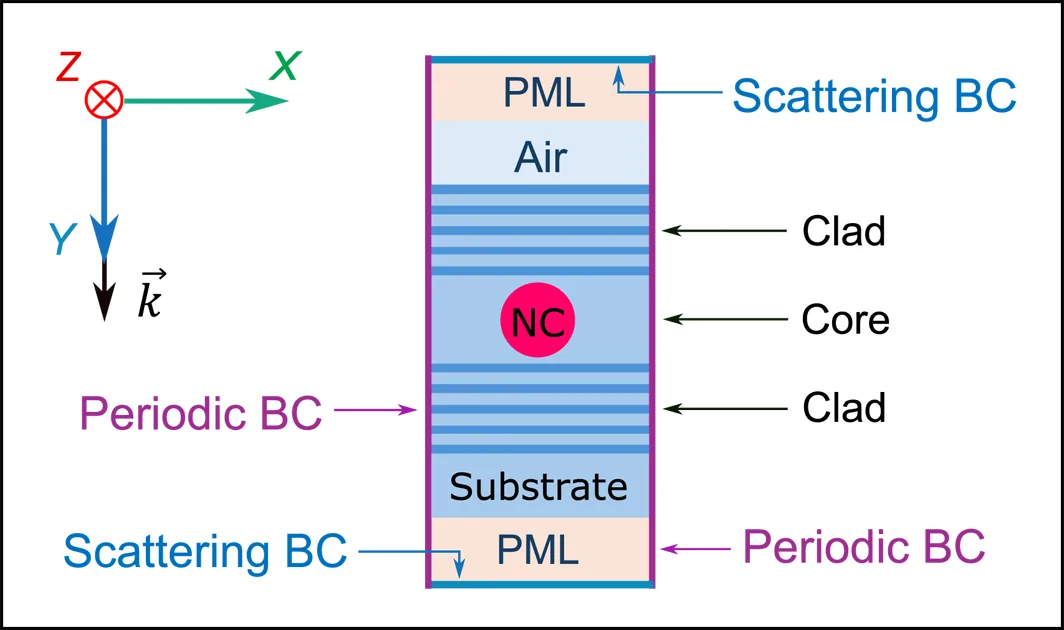
Simulation domain of the ENZ Bragg cavity loaded with a periodic array of infinitely long silicon cylinders of radius R=150nm. The domain is enclosed by periodic and scattering boundary conditions (BCs).

The choice of materials in the optical photonic doping platform is governed by three key considerations: (i) Sufficient refractive‐index contrast to support Mie resonances in the NCs, where higher contrast enables reduced inclusion size and enhanced Purcell factors, whereas lower‐index inclusions require larger radii or size parameter to exceed the threshold value for the onset of a Mie resonance, potentially increasing mode volume; (ii) minimization of material absorption and scattering losses, which directly limit the achievable Q‐factor and photonic‐doping efficiency and depend on both intrinsic material properties and fabrication‐induced imperfections; and (iii) compatibility with established fabrication processes, as exemplified by CMOS‐compatible platforms such as Si/${SiO}_{2}$/SiN, whereas alternative high‐index materials (e.g., Ge or GaP) may be employed within suitable low‐loss spectral ranges.

### 3.1 Excitation of Transverse Electric Resonances

Transverse electric (TE) resonances arise when the electric field component of the incident wave is oriented perpendicular to the NC axis. Figure 5 presents the transmittance spectra of a cavity, both with and without the NC array in the core. The cavity consists of a half‐wave ${\text{SiO}}_{2}$ core with refractive index of $n_{SiO_{2}}=1.44+i10^{-6}$ [56], sandwiched between two Bragg mirrors, each composed of 10 pairs of quarter‐wave cladding layers made of ${\text{SiO}}_{2}$ and SiN $\left(n_{\text{SiN}}=2+i10^{-7}\right)$ [57]. The cutoff wavelength of the cavity is set to $\lambda_{c}=1500\;nm$. The system is doped with an array of silicon NCs with radii R=150nm and refractive index $n_{\text{Si}}=3.5+i1.38\times 10^{-13}$ [58], positioned at the center of the core. The periodicity of the array is p=500nm and p=700nm in Figure 5b,c, respectively.

**FIGURE 5:**
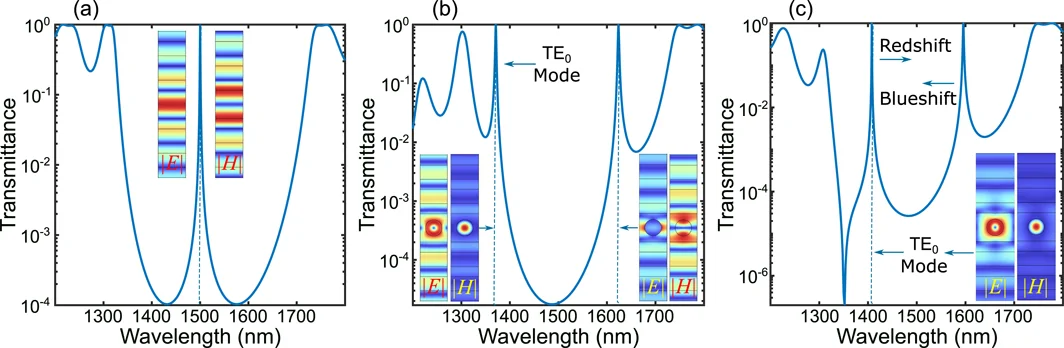
Transmittance spectra and corresponding field profiles. (a) Bare Bragg cavity (cutoff wavelength $\lambda_{c}=1500\;nm$) consisting a half‐wave ${\text{SiO}}_{2}$ core, sandwiched between 10 pairs of quarter‐wave cladding layers of ${\text{SiO}}_{2}$ and SiN. (b,c) The same cavity loaded with arrays of infinitely long silicon NCs, placed at the core center, with lattice periods (b) p=500nm and (c) p=700nm. All structures are illuminated by a normally incident plane wave (along y‐axis) with the magnetic field H along z‐axis (TE, $H_{z}$).

Figure 5a shows the transmittance spectrum and field profiles of the bare cavity mode, with a transmission peak very close to the cutoff wavelength, $\lambda_{c}=1500\;nm$. The electric field is mainly concentrated to the center of the core, whereas the magnetic field is localized at the core boundaries. Introduction of the NC array (Figure 5b) turns the bare cavity mode into two resonant modes within the Bragg stopband, one at a longer wavelength and the other at a shorter wavelength relative to the bare‐cavity cutoff. As demonstrated later, these correspond to NZI modes of the structure. The higher‐energy mode, located at λ=1371nm, is dominantly confined inside the cylinders and can be identified as the Mie ${\text{TE}}_{0}$‐like mode with an enhanced Q‐factor of Q=626, associated with the magnetic dipole resonance of the Mie coefficient $a_{0}$. The lower‐energy NZI mode, at λ=1623.5nm with a Q‐factor of Q=607, is characterized by distinct electric and magnetic field distributions that are mainly localized outside the cylinders. Increasing the periodicity of the array preserves the general field distribution patterns of both the high‐ and low‐energy modes. However, the Q‐factors of the modes increase, and as shown in Figure 5c, the lower‐energy mode exhibits a blue shift while the higher‐energy mode undergoes a red shift, which can extend beyond the cutoff wavelength of the bare cavity (see Figure 6).

**FIGURE 6:**
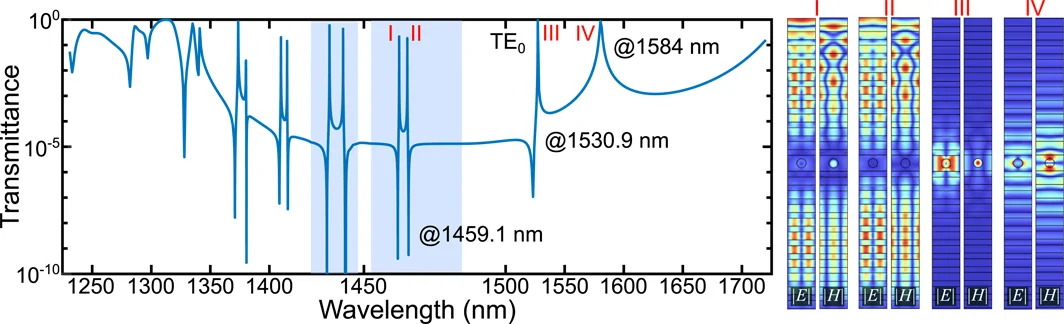
Transmittance spectrum and field profiles of the resonant modes in the same Bragg cavity as in Figure 5b, with a period of p=900nm, under TE plane–wave illumination. To visually resolve closely spaced resonances, two wavelength intervals highlighted in blue are displayed with nonuniform horizontal scaling: The regions 1335–1345nm and 1355–1365nm are expanded by factors of 4 and 8, respectively, relative to the rest of the spectrum.

By increasing the periodicity to p=900nm, the Q‐factor of the ${\text{TE}}_{0}$‐like mode rises to $Q=2.32\times 10^{4}$, which is about $10^{3}$ times higher than for corresponding free‐space array and over 14 times higher than that of the bare cavity. As shown in Figure 6, additional resonant structural modes also appear, with electromagnetic fields predominantly distributed within the Bragg layers but exhibiting exceptionally high Q‐factors; for example, $Q=4.87\times 10^{5}$ at λ=1459.1nm, even higher than that of the confined ${\text{TE}}_{0}$‐like mode.

Comparison of the magnetic field distribution of the ${\text{TE}}_{0}$‐like mode with that of the bare‐cavity mode (Figure 5a) reveals that enhanced field localization, together with the significant increase in Q‐factor, produces magnetic hot spots within the NCs, which is one of the key features of photonically doped ENZ media [16].

In these structures, although the period of the doped NC array modulates the transmission spectrum, leading to shifts in the resonance position and changes in bandwidth, the array periodicity by itself, when decoupled from the cavity, does not support the formation of narrowband resonances. This indicates that the emergence of high‐Q resonances is primarily governed by the ENZ cavity rather than by collective lattice effects. This conclusion is supported by Figure 7, which compares the transmittance of a TE‐polarized plane wave through a single NC with that through arrays of identical NCs with varying periodicities. The curves show that arranging the NCs into an array causes the transmission peak to broaden and shift to shorter wavelengths, with the effect becoming more pronounced at smaller periods.

**FIGURE 7:**
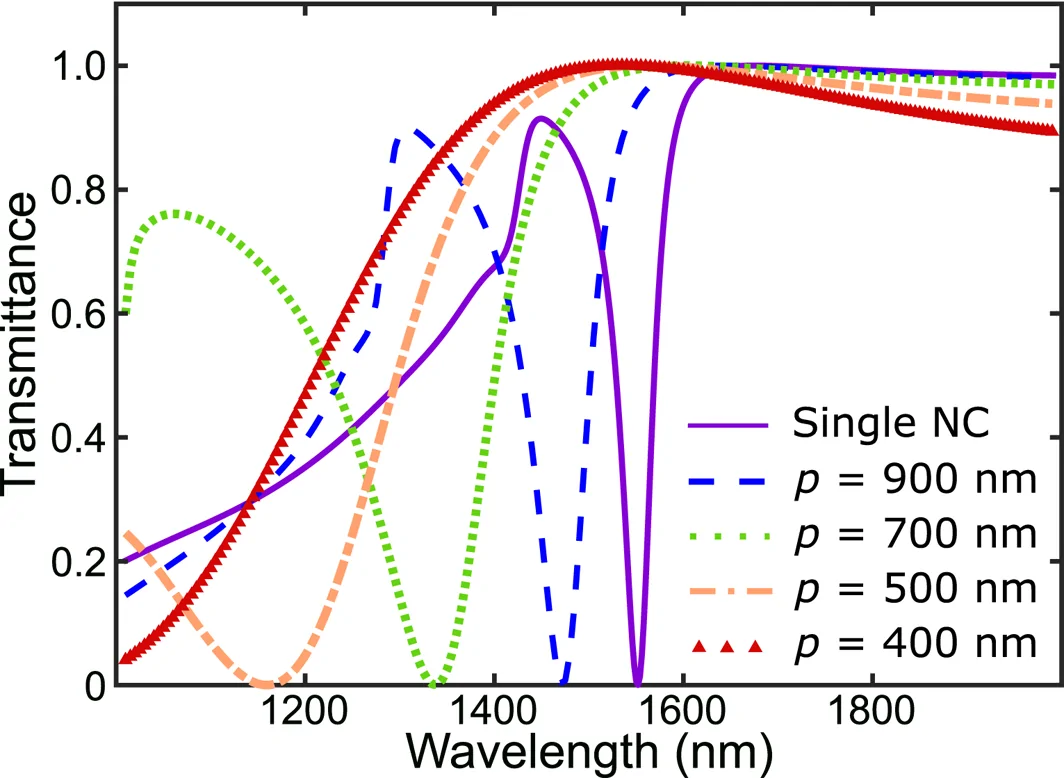
Transmittance spectra of a single NC of radius R=150nm (solid curve) and index $n_{\text{Si}}$ in a ${\text{SiO}}_{2}$ matrix as well as arrays of such NCs with periods varying between 400 − 900 nm.

#### 3.1.1 PMC and EMNZ Features in Photonically Doped Cavities

To demonstrate the PMC and EMNZ features by photonic doping of Bragg cavities, the same structure as in Figure 5b is considered, but with increased number of Bragg layers to N=14 pairs of quarter‐wave layers. The transmittance spectrum of the structure is shown in Figure 8a, together with the corresponding field profiles of the ${\text{TE}}_{0}$‐like mode at λ=1372.38nm. We also computed the dispersive effective refractive index (using COMSOL's mode analysis module), along with the effective relative permittivity and permeability around this mode, as presented in Figure 8b.

**FIGURE 8:**
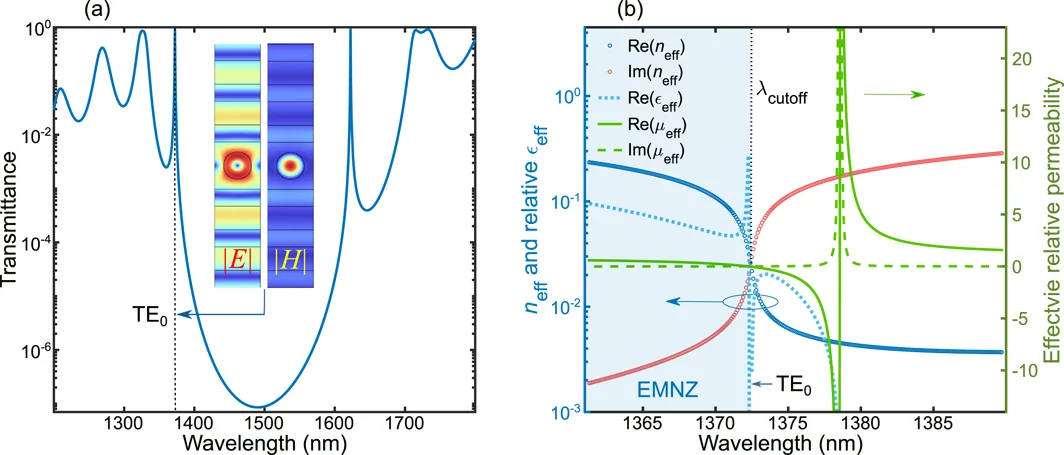
(a) Transmittance and resonant‐mode field distribution corresponding to a Bragg cavity with N=14 pairs of cladding layers on each side of the half‐wave core and doped with an array of cylindrical NCs of radius R=150nm having a period of p=500nm. (b) Calculated effective refractive index as well as effective relative permittivity and permeability around the wavelength λ=1372.38nm corresponding to the ${\text{TE}}_{0}$‐like resonant mode in the same NC‐doped Bragg cavity.

In order to consistently define the effective magnetic permeability of a periodic array of inclusions, we follow the homogenization procedure introduced by Silveirinha and Engheta [7]. For a periodic Bloch mode, the microscopic electric and polarization current density fields have the form as follows: [59]

(8) $$E\left(r\right)=\tilde{E}\left(r\right)\;e^{ik_{B}\cdot r},\;J\left(r\right)=\tilde{J}\left(r\right)\;e^{ik_{B}\cdot r},$$

where $\tilde{E}$ and $\tilde{J}$ are periodic functions over the unit cell and $k_{B}$ is the Bloch wavevector. Accordingly, the microscopic magnetic dipole moment per unit length is written as follows: [59]

(9) $$m_{z}\;=\;\frac{1}{2}\int \int_{\text{inc}}\left[\left(x-x_{c}\right)J_{y}-\left(y-y_{c}\right)J_{x}\right]\;e^{-ik_{B}\cdot r}\;dA,$$

where $J_{x}=-i\omega \left(\varepsilon -\varepsilon_{\text{host}}\right)E_{x}$ and $J_{y}=-i\omega \left(\varepsilon -\varepsilon_{\text{host}}\right)E_{y}$ are the polarization current densities inside the inclusion, and $\left(x_{c},y_{c}\right)$ is the centroid of the inclusion cross‐section, included to avoid origin dependence. The corresponding magnetization is normalized over the whole unit cell area,

(10) $$M_{z}=\frac{m_{z}}{A_{\text{cell}}},\;A_{\text{cell}}=\int \int_{\text{cell}}dA.$$

The dephasing factor $e^{-ik_{B}\cdot r}$ is also used consistently in the evaluation of all other cell‐averaged quantities, such as $\langle H_{z}\rangle$, ensuring that the retrieved effective permeability $\mu_{\text{eff}}$ is invariant under translations of the unit cell and fully consistent with the Floquet–Bloch homogenization theory. However, because the mode under consideration is an NZI mode, with effective index close to zero, the Bloch wavevector is negligible and the dephasing factor can be safely ignored.

Because the direct cell average of the magnetic field $\langle H_{z}\rangle$ contains contributions from both the applied field and the self‐field of the induced dipole, a corrected bulk magnetic field is introduced as follows: [7]

(11) $$H_{\text{bulk}}=\langle H_{z}\rangle -\frac{m_{z}}{A_{\text{cell}}}.$$

The constitutive relation can then be written as follows:

(12) $$B=\mu_{0}\langle H_{z}\rangle =\mu_{\text{eff}}H_{\text{bulk}},$$

which leads to the following expression for the effective relative permeability:

(13) $$\mu_{r}=\frac{\mu_{\text{eff}}}{\mu_{0}}=\frac{\langle H_{z}\rangle }{H_{\text{bulk}}}.$$

In Figure 8b, the real and imaginary parts of the effective index of refraction are shown by the dark‐blue and red‐circle curves, respectively. The real and imaginary parts of the effective relative permeability are plotted as green curves, whereas the effective relative permittivity, calculated from the relation $\varepsilon_{\text{eff}}=n_{\text{eff}}^{2}/\mu_{\text{eff}}$, is shown by the dotted light‐blue curve.

The ${\text{TE}}_{0}$‐like mode resonates at λ=1372.38nm, in the close vicinity of the cutoff. The PMC‐like response appears at the wavelength where the effective permeability (solid green) exhibits a pole $\left(\mu_{r}\to \infty \right)$, corresponding to a high‐impedance condition. The EMNZ region, corresponding to wavelengths shorter than the cutoff wavelength, is highlighted in blue. In this region, both the effective permittivity and permeability are positive and smaller than unity. For wavelengths longer than the cutoff wavelength, the signs of the effective permittivity and permeability are opposite, and consequently the wave cannot propagate in this spectral range, as indicated by the rapid growth of the imaginary part of the effective index (red‐circle curve).

It is interesting to note that, although the microscopic magnetic dipole moment $m_{z}$ and consequently the ${\text{TE}}_{0}$‐like mode exhibits a resonance at λ=1372.38nm, the effective relative permeability diverges at a slightly longer wavelength. This red shift follows directly from the retrieval formula

(14) $$\mu_{r}=\frac{\langle H_{z}\rangle }{\langle H_{z}\rangle -M_{z}},\;M_{z}=\frac{m_{z}}{A_{\text{cell}}}.$$

Although $M_{z}$ peaks at the intrinsic magnetic‐dipole‐like resonance of the inclusions, the pole of $\mu_{r}$ occurs when the bulk field in the denominator, $H_{\text{bulk}}=\langle H_{z}\rangle -M_{z}$, vanishes. The relative phase and dispersive behavior of $\langle H_{z}\rangle$ therefore shift the divergence of $\mu_{r}$ to longer wavelengths than the maximum of $M_{z}$.

### 3.2 Excitation of Transverse Magnetic (TM) Resonances

The TM resonances of the doped Bragg cavity are excited by illumination with a normally incident plane wave with its electric field polarized along the z‐axis. The transmittance and field profiles of ${\text{TM}}_{1}$‐like modes are shown in Figure 9. For a period of p=500nm only the ${\text{TM}}_{1}$‐like mode exists, and is confined inside the NCs (Figure 9a). When the period is increased to p=750nm, an additional mode emerges at shorter wavelengths (Figure 9b). The fields of this mode are primarily confined to the regions between the NCs. When the period is further increased to p=900nm, both modes undergo a redshift, approach each other, and exhibit enhanced Q‐factors (Figure 9c). At the same time, other modes also appear at shorter wavelengths, with their fields predominantly distributed in the Bragg mirror layers rather than within the cavity core.

**FIGURE 9:**
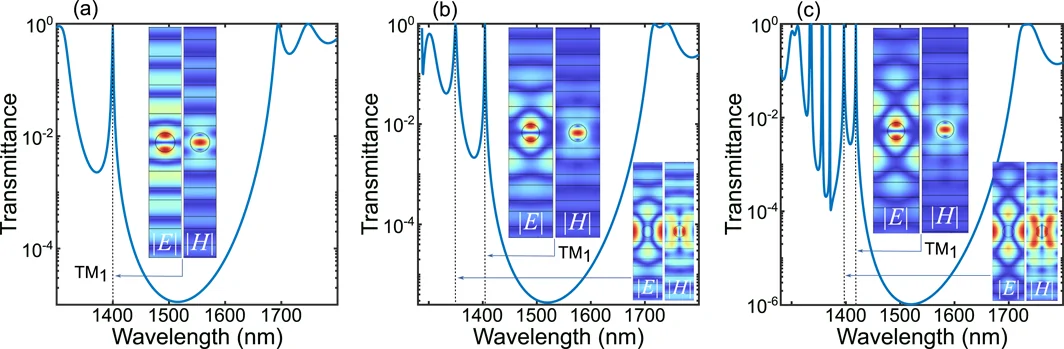
Transmittance and the resonant‐mode field distribution of a Bragg cavity with the same parameters as in Figure 5, loaded with an array of NCs (R=150nm), for periods of (a) p=500nm, (b) p=750nm, and (c) p=900nm. All structures are illuminated by a normally incident plane wave (along y‐axis) with the E‐field along z‐axis (TM, $E_{z}$).

In contrast to TE resonances, TM excitation primarily induces an electric dipole along the z axis, with no pronounced magnetic response. Consequently, although the TM‐like resonances shown in Figure 9 correspond to NZI TM‐like Bragg–Mie modes, they are not associated with effective‐medium EMNZ states within a homogenization framework.

### 3.3 Tunability of the Bragg–Mie Modes Beyond the Size Parameter

In addition to the conventional size parameters $x_{i}$ and $x_{e}$, which depend on wavelength, particle size, and the refractive indices of the particle and surrounding medium [52], the spectral and spatial properties of the Bragg–Mie modes can be further tuned by external and structural parameters such as the cutoff wavelength (set by the core thickness), the lattice period of the dopant array, and the angle of incidence (AoI). These parameters enable tunability beyond what is achievable with the size parameter alone, providing additional degrees of freedom for engineering the spectral position and field confinement of the resonances. For instance, this approach allows alignment of the cavity modes with the magnetic dipole resonances of doped atoms or quantum dots, facilitating the enhancement or selective excitation of their intrinsic magnetic dipole transitions.

Figure 10 illustrates this tunability for both the ${\text{TE}}_{0}$‐like (Figure 10a) and ${\text{TM}}_{1}$‐like (Figure 10b) modes by showing the transmittance of a doped Bragg cavity with the same parameters as in Figure 5, for p=500nm and different bare‐cavity cutoff wavelengths of $\lambda_{c}=1450\;nm$, 1500nm, and 1550nm. For both modes, the resonance redshifts for larger cutoff wavelengths.

**FIGURE 10:**
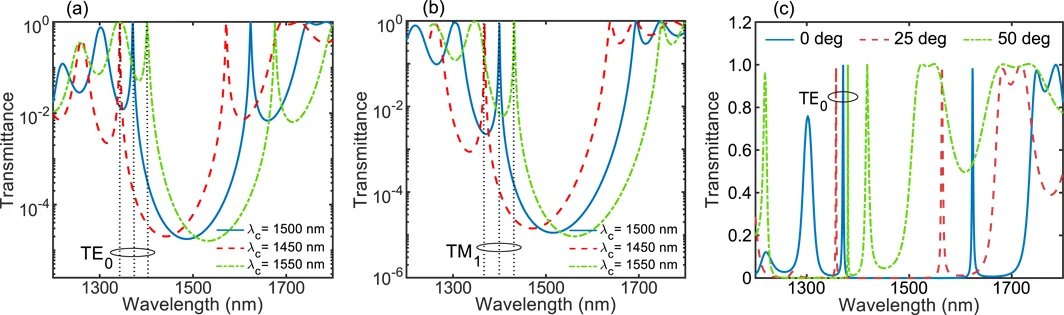
Transmittance spectra of a doped Bragg cavity with period p=500nm for (a) ${\text{TE}}_{0}$‐like and (b) ${\text{TM}}_{1}$‐like modes at different cutoff wavelengths $\lambda_{c}=1450\;nm$ (red dashed), 1500nm (blue solid), and 1550nm (green dotted). (c) TE‐polarized transmittance of the same structure at $\lambda_{c}=1500\;nm$ for incidence angles of 0° (solid), 25° (dashed), and 50° (dash‐dotted).

Figure 10c shows the TE‐polarized transmittance of the same structure at $\lambda_{c}=1500\;nm$ for increasing AoI. The lower‐ and higher‐energy modes shift and move closer together, illustrating an angle‐tunable response; the lower‐energy branch blue‐shifts across the range, whereas the higher‐energy branch shows a weaker non‐monotonic shift at larger angles.

Figure 11 shows the TM‐polarized transmittance of the same structure as in Figure 10, but with p=750nm and AoIs of 0°, 5°, and 15°. As seen in Figure 10a, for non‐zero AoIs, new resonances emerge with markedly high Q‐factors, particularly at smaller angles (e.g., $Q\approx 1.5\times 10^{4}$ for AoI =5°, compared to Q≈700 for the ${\text{TM}}_{1}$‐like mode). This resonance is structurally similar to the ${\text{TM}}_{1}$‐like mode but exhibits a 90° rotated field distribution. Upon increasing AoI, the mode redshifts while the ${\text{TM}}_{1}$‐like mode gradually vanishes. As illustrated in Figure 11b for an AoI of 15°, additional resonances also appear at shorter wavelengths, with their fields predominantly distributed in the Bragg mirror layers rather than in the cavity core.

**FIGURE 11:**
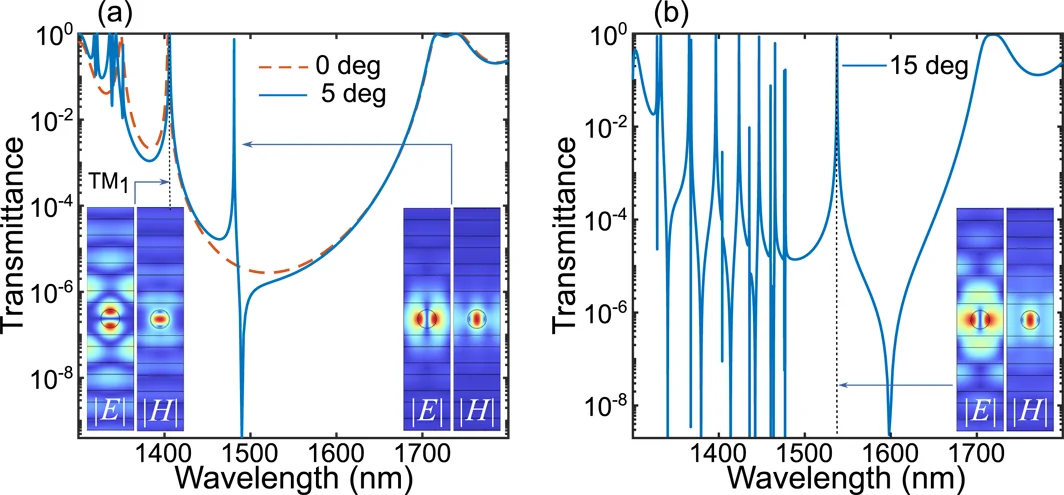
TM‐polarized transmittance of a Bragg cavity with N=10 pairs of cladding layers on each side of a half‐wave core, designed for a cutoff wavelength of 1500nm, and loaded with an array of cylindrical NCs (R=150nm) with period p=750nm. (a) Results for incidence angles of 0° (dashed curve) and 5° (solid curve). (b) Results for an incidence angle of 15°.

The modes discussed above—including the TE‐ and TM‐like resonances confined inside the NCs, the modes localized between the NCs, and the high‐Q resonance emerging at non‐zero AoI in Figure 11a—can all be identified as high‐FoM NZI modes of the doped Bragg cavity. This is confirmed by the dispersion curves shown in Figure 12a in the wavelength range of 1300–1650 nm, together with their corresponding transmission peaks presented in Figure 12b.

**FIGURE 12:**
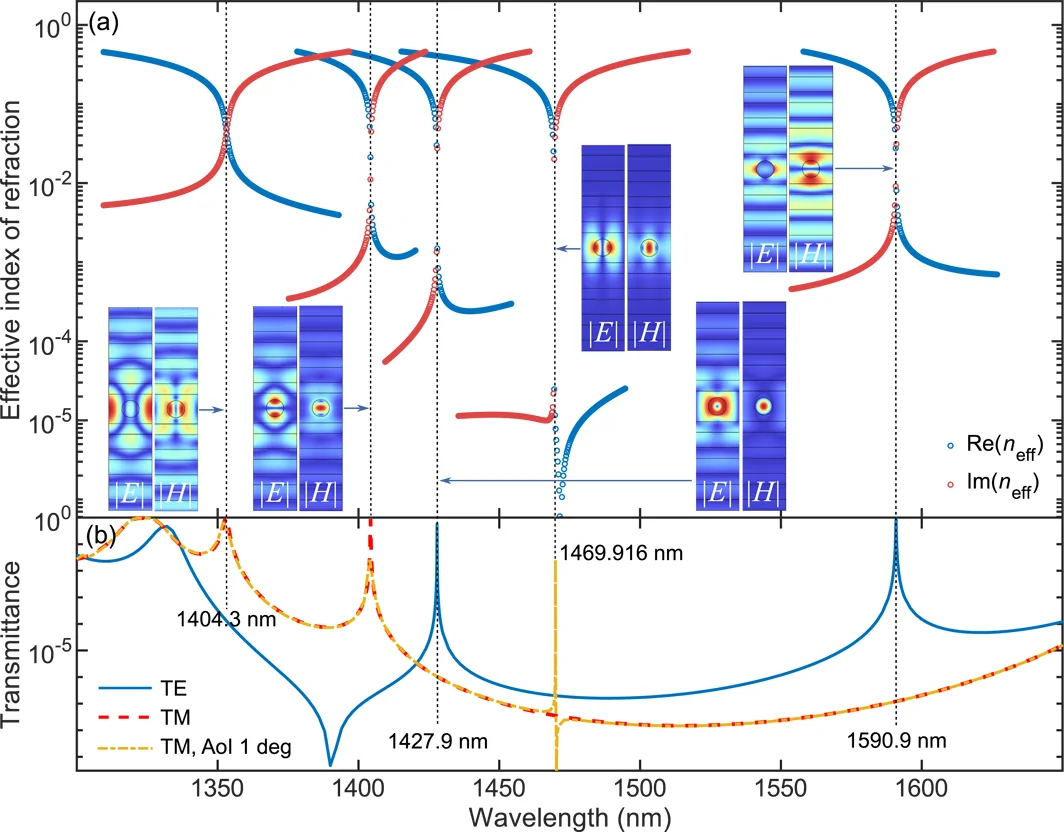
(a) Real and imaginary parts of effective refractive indices of TE and TM modes, together with their corresponding field distributions, in a Bragg cavity with N=14 pairs of cladding layers on each side of a half‐wave core, loaded with an array of cylindrical NCs, with a period of p=750nm. (b) Transmission spectrum of the same cavity for TE (solid curve) and TM (dashed/dotted curves) modes.

### 3.4 Magnetic Purcell Enhancement in NC‐Doped ENZ Cavity

To quantify the spontaneous‐emission enhancement in the NC‐doped ENZ Bragg cavity, we evaluate the Purcell factor (PF) and compare it with that of a single NC or an array of NCs embedded in an infinite homogeneous medium. The formulation follows the Green‐tensor approach previously introduced for two‐dimensional (2D) photonic systems [60], extended here to account for both electric and magnetic emitters by means of the corresponding electric and magnetic Green functions.

The Purcell factor is defined as the ratio of the power emitted by a source in the structure to that in a homogeneous background medium. For a current source J located at $r_{0}$, the emitted power is given by $P=\left(1/2\right)R\left\{J^{*}\cdot E\left(r_{0}\right)\right\},$ where $E\left(r_{0}\right)$ is the electric field at the source position. This quantity is also proportional to the imaginary part of the dyadic Green tensor evaluated at the source location, corresponding to the local density of electromagnetic states [61]:

(15) $$\frac{\Gamma \left(\omega \right)}{\Gamma_{0}\left(\omega \right)}\propto J^{*}\cdot I\left[G\left(r_{0},r_{0}\right)\right]\cdot J.$$

Thus it can be shown (Supporting Information S1: Section C) that the PF is obtained directly from the electric field at the source position normalized by the field produced by the same line source in the homogeneous background:

(16) $$F_{P}\left(\omega \right)=\frac{R\left\{E_{z}\left(r_{0},\omega \right)\right\}}{R\left\{E_{z,0}\left(r_{0},\omega \right)\right\}}.$$

A parallel derivation applies to a magnetic line source oriented along z (Supporting Information S1: Section C), resulting in the magnetic PF in terms of the magnetic field at the source location in the structure and in the homogeneous background medium:

(17) $$F_{P,m}\left(\omega \right)=\frac{R\left\{H_{z}\left(r_{0},\omega \right)\right\}}{R\left\{H_{z,0}\left(r_{0},\omega \right)\right\}}.$$

Therefore, in our 2D cavity, the PF is evaluated by exciting the structure with either an electric or a virtual magnetic line current placed at the center of an NC in the core, oriented along z. The PF is then obtained from the above expressions by computing the real part of the corresponding z‐component of the field at the source position and normalizing to its free‐space value.

Figure 13a compares the calculated PF spectra at the center of a single silicon NC in free space (dashed line), an array of 25 NCs in free space (dashed‐dotted line), and the same NC array embedded in the Bragg cavity (solid line). The embedded array exhibits a nearly 35‐fold magnetic Purcell enhancement (PF=833) at λ=1429.6nm relative to the isolated NC or free‐space array. The corresponding electric and magnetic field distributions at resonance are shown in Figure 13b,c. In these simulations, the Bragg cavity consists of N=14 cladding–layer pairs on each side of a half‐wave core, loaded with an array of 25 silicon NCs of radius R=150nm with period p=750nm. The cavity width is W=18.75μm. The NZI Bragg–Mie mode and the associated Purcell enhancement are retained under vertical displacement of the array within the core, although the PF exhibits a moderate reduction. For example, a vertical shift of 50nm in the structure of Figure 13 reduces the maximum PF from 833 to 676, whereas the mode remains intact despite the resulting vertical asymmetry of the structure. This indicates that the response of the Bragg‐based photonic doping platform is not strictly position‐invariant but remains robust to moderate positional perturbations of the NC array.

**FIGURE 13:**
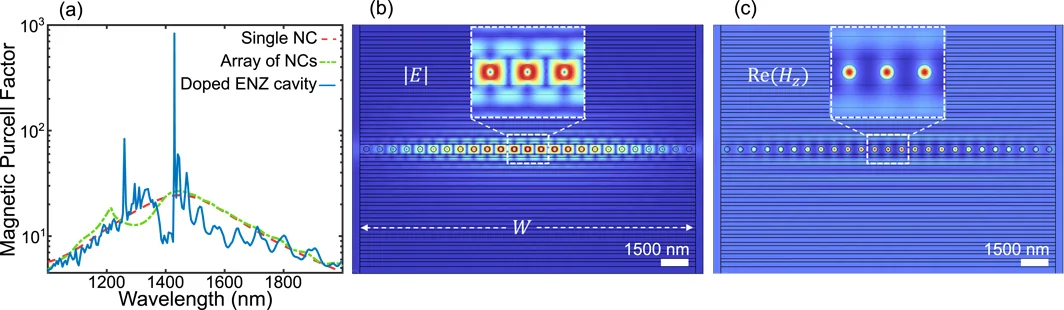
(a) Magnetic PF for a single NC in free space, an array of 25 NCs in free space, and the same NC array embedded in the Bragg cavity. (b) Electric‐field magnitude |E| and (c) magnetic‐field component $Re\left(H_{z}\right)$ at the resonant wavelength. The Bragg cavity comprises of N=14 cladding‐layer pairs on each side of a half‐wave core and is loaded with an array of 25 NCs (R=150nm, $n=n_{\text{Si}}$, p=750nm).

Interestingly, a significant enhancement of the magnetic PF can be achieved by optimizing the cavity geometry. For instance, increasing the core thickness of the structure in Figure 13 to 868nm, and therefore shifting the fundamental ENZ resonance of the bare cavity to λ=1690nm, results in more than one order‐of‐magnitude improvement in the magnetic PF. In this configuration, as shown in Figure 14, the NZI Bragg–Mie mode redshifts to λ=1508.22nm and exhibits a magnetic PF exceeding $1.8\times 10^{4}$.

**FIGURE 14:**
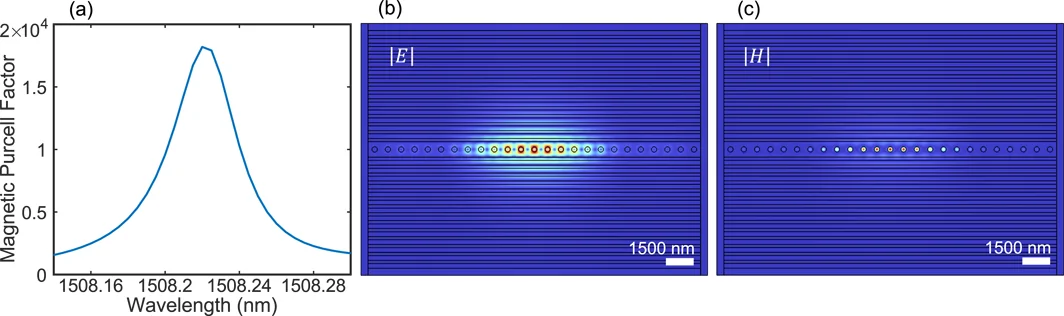
(a) Magnetic PF spectrum for the same structure and excitation method as in Figure 13, but optimized with increasing core thickness from 520.83nm − 868nm. (b) The electric and (c) magnetic‐field distributions of the NZI mode at the resonance wavelength of λ=1508.22nm.

This enhancement can be attributed to the interplay of three mechanisms. First, increasing the core thickness in such a Fabry–Pérot‐type cavity leads to an increase in the cavity Q‐factor due to the longer photon round‐trip time, whereas the effective mode volume remains largely governed by the localized NZI hybrid mode. Second, as the core thickness increases, the NC inclusions become less influenced by the nearby core boundaries, allowing the hybrid mode to evolve toward a more Mie‐like character. As a result, the electromagnetic field becomes more strongly confined within the NCs, leading to a reduction in the effective mode volume. Third, retuning of the cavity–dopant hybridization can modify the effective impedance contrast with the surrounding medium, enhancing the lateral confinement of the NZI mode and producing a more localized field antinode within the cavity, as observed in Figure 14b,c compared to Figure 13b,c. The combined effect of increased Q and improved field localization results in the observed PF enhancement.

As discussed in Supporting Information S1: Section D, with further increase in the core thickness, although the cavity Q continues to grow, the spatial overlap between the bare‐cavity ENZ mode and the NC Mie mode progressively decreases. Consequently, the hybrid mode becomes increasingly delocalized across the enlarged core region between the Bragg mirrors, leading to a larger effective mode volume compared to the optimized case in Figure 14, where the field is predominantly confined within or in the immediate vicinity of the NCs. A detailed discussion of the role of the ENZ condition in PF enhancement is also provided in Supporting Information S1: Section D.

The electric Purcell enhancement in NC‐doped Bragg cavities can be investigated for NZI TM‐polarized modes by exciting the structure with electric line currents placed at the electric hot spots of the modes (see Supporting Information S1: Section E). It should be noted that the use of electric or virtual magnetic line currents is purely a computational tool to excite the corresponding TM or TE modes; in practice, the TE‐ and TM‐polarized modes can be excited by plane‐wave illumination with the appropriate polarization.

## 4 Concluding Remarks

It is shown that photonic doping of ENZ Bragg cavities with periodic arrays of dielectric NCs provides a robust route to engineer high‐Q NZI modes with well‐controlled electric and magnetic character. On the analytical side, we demonstrated that embedding Mie resonators in an ENZ background generically suppresses radiative losses and narrows both electric and magnetic multipolar resonances for cylindrical and spherical inclusions. Numerically, we established that near‐cutoff all‐dielectric Bragg cavities doped with resonant silicon NC arrays support coupled Bragg–Mie modes with effective EMNZ response, Q‐factors on the order of $10^{4}$, and magnetic‐dipole Purcell enhancements exceeding $1.8\times 10^{4}$ for a 18μm‐scale doped structure, far beyond what is achievable with isolated particles or undoped cavities.

The high‐Q NZI modes can be selectively excited and tuned through structural and external parameters such as lattice period, cutoff wavelength, and AoI. This tunability enables access to spectrally isolated, predominantly single‐multipole resonances and to controlled interference between electric‐ and magnetic‐dipole‐like channels. The localized and high‐Q resonances of the NCs provide large LDOS enhancements for both electric and magnetic emitters, with well‐defined multipolar selectivity.

Beyond the specific configurations considered here, the concept of ENZ photonic doping is readily extendable to alternative dopant geometries (Supporting Information S1: Section G), higher‐order cavity modes as well as to active and nonlinear materials. From a practical standpoint, the proposed photonic doping platform is compatible with established all‐dielectric fabrication and characterization techniques. Realistic material losses were explicitly accounted for (Supporting Information S1: Section F), showing that even for material losses corresponding to $Im\left(n\right)∼10^{-4}$, the system maintains Q‐factors and Purcell enhancements on the order of $10^{3}$, demonstrating strong robustness against dissipative and structural imperfections. CMOS‐compatible platforms such as Si/${SiO}_{2}$/SiN provide a viable implementation route (Supporting Information S1: Section G), whereas alternative high‐index III–V semiconductor materials may be employed within appropriate low‐loss spectral windows. Low‐loss dielectric materials such as ${\text{Ta}}_{2}O_{5}$ remain well suited for Bragg reflectors, where ultra‐high reflectivities have been demonstrated [62]. Related Bragg‐cavity and metasurface‐integrated structures have already been experimentally realized [63, 64, 65, 66]. Standard optical characterization methods including transmission/reflection spectroscopy for extracting cavity Q‐factors and time‐resolved photoluminescence for evaluating spontaneous emission enhancement are directly applicable to the present platform (Supporting Information S1: Section H). Together, these considerations indicate that the proposed ENZ photonic doping concept is experimentally accessible with current nanofabrication and measurement capabilities.

The proposed platform is envisioned to serve as a versatile building block for low‐threshold and multipolar‐selective lasing, spectroscopy of magnetic dipole or higher‐order transitions, enhanced magnetically driven nonlinear processes, and engineered quantum light–matter interactions.

## Author Contributions

**Ali Panahpour:** conceptualization, investigation, methodology, validation, formal analysis, visualization, writing – original draft. **Jussi Kelavuori:** validation, writing – review and editing. **Mikko Huttunen:** funding acquisition, writing – review and editing, validation, supervision, resources.

## Supporting information


Supporting Information S1

## Data Availability

The data that support the findings of this study are available from the corresponding author upon reasonable request.

## Endnotes

^1^
Extensions of the photonic doping concept to other electromagnetic media are possible, for example in EMNZ photonic crystals [17], where the underlying physical mechanisms differ from those in ENZ media. However, the present study is restricted to photonic doping of ENZ media.
