# Supplementary material for: Photonic Doping of Epsilon‐Near‐Zero Bragg Microcavities
Source: Nanophotonics. 2026 Jun 26;15(13):e70186. doi: 10.1002/nap2.70186 (PMC13642544; doi:10.1002/nap2.70186)
Supplement: Supplementary file 1 [file NAP2-15-e70186-s001.pdf]

# Supporting Information for

# Photonic Doping of Epsilon-Near-Zero Bragg Microcavities

Ali Panahpour, Jussi Kelavuori, and Mikko Huttunen*

*Photonics Laboratory, Physics Unit, Tampere University, FI-33014 Tampere, Finland*

E-mail: mikko.huttunen@tuni.fi

## Section A: Mie Resonances of Cylindrical Inclusions in ENZ Media

**Polarization and mode conventions.** Throughout this work, the axis of the nanocylinder (NC) dopant is taken along the $z$-direction. We adopt the standard two-dimensional (2D) scattering convention for infinitely long cylinders, in which the fields are decomposed according to the axial field component. Specifically, *TE polarization* (transverse electric to the cylinder axis) corresponds to $E_z = 0$ and $H_z \neq 0$, whereas *TM polarization* (transverse magnetic to the cylinder axis) corresponds to $H_z = 0$ and $E_z \neq 0$. Following the notation of Kerker and Bohren & Huffman, the cylindrical Mie coefficients $a_n$ and $b_n$ describe, respectively, the TE ($H_z$) and TM ($E_z$) polarization channels. When referring to $\mathrm{TE}_n$ or $\mathrm{TM}_n$ modes of the cylinder, the subscript $n$ denotes the azimuthal order of the cylindrical harmonic, and the mode label specifies the polarization channel determined by the nonzero axial field component. This definition differs from waveguide-style TE/TM conventions based on propagation direction.

We consider the Mie coefficients corresponding to TE modes of an infinite lossless cylinder:

$$a_n = \frac{x_i J_n(x_i) J_n'(x_e) - x_e J_n(x_e) J_n'(x_i)}{x_i J_n(x_i) H_n^{(1)'}(x_e) - x_e H_n^{(1)}(x_e) J_n'(x_i)}. \tag{1}$$

The extinction coefficient is proportional to the real part of $a_n$, which can be written in terms of the real numerator ($N$) and complex denominator ($D$) of the coefficient as

$$\Re(a_n) \approx \Re\left(\frac{1}{D}\right) N = \frac{\Re(D) N}{\Re(D)^2 + \Im(D)^2}, \tag{2}$$

where $\Re(D)$ and $\Im(D)$ are the real and imaginary parts of $D$. We show that in the ENZ regime, the real part of the Mie coefficients $a_n$ (or $b_n$) behaves like a Lorentzian function normalized to unity at resonance,

$$L(\omega) = \frac{\gamma^2}{\Delta\omega^2 + \gamma^2}. \tag{3}$$

In this representation, $\Im(D)$ vanishes at the resonance frequency and thus represents the resonance detuning $\Delta\omega$, while $\Re(D)$ approaches zero as the surrounding refractive index tends to zero, corresponding to the narrowing Lorentzian linewidth $\gamma$.

First, for $n = 0$, using the small-argument approximations

$$H_0^{(1)}(x_e) \approx \left(1 - \frac{x_e^2}{4}\right) + i \cdot \frac{2}{\pi}\left(\ln\left(\frac{x_e}{2}\right) + \gamma\right), \tag{4}$$

$$H_0^{(1)\prime}(x_e) \approx -\frac{x_e}{2} + i \cdot \left(-\frac{2}{\pi x_e}\right), \tag{5}$$

the real and imaginary components of the denominator are obtained as

$$\Re(D) = -\frac{x_i x_e}{2} J_0(x_i) - x_e J_0'(x_i)\left(1 - \frac{x_e^2}{4}\right), \tag{6}$$

$$\Im(D) = -\frac{2x_i J_0(x_i)}{\pi x_e} - \frac{2x_e J_0'(x_i)}{\pi}\left(\ln\left(\frac{x_e}{2}\right) + \gamma\right). \tag{7}$$

Using the small-argument approximations for the Bessel function,

$$J_0(x_e) \approx 1 - \frac{x_e^2}{4}, \tag{8}$$

$$J_0'(x_e) \approx -\frac{x_e}{2}, \tag{9}$$

the simplified expression for the numerator becomes

$$N \approx -\frac{1}{2} x_i x_e J_0(x_i) - x_e J_0'(x_i)\left(1 - \frac{x_e^2}{4}\right). \tag{10}$$

Therefore, when $x_e \to 0$, the resonance condition is obtained from the (7) as $J_0(x_i) = 0$, and for the resonance width we have from (6):

$$\gamma_0 \propto -x_e J_0'(x_i)\left(1 - \frac{x_e^2}{4}\right), \tag{11}$$

which tends to zero as $x_e \to 0$.

For $n > 0$, using the small-argument approximations

$$H_n^{(1)}(x_e) \approx \frac{1}{n!}\left(\frac{x_e}{2}\right)^n - \frac{1}{(n+1)!}\left(\frac{x_e}{2}\right)^{n+2} + i \cdot \frac{(n-1)!}{\pi}\left(\frac{2}{x_e}\right)^n, \tag{12}$$

$$H_n^{(1)\prime}(x_e) \approx \frac{n}{2n!}\left(\frac{x_e}{2}\right)^{n-1} - \frac{(n+2)}{2(n+1)!}\left(\frac{x_e}{2}\right)^{n+1} - i \cdot \frac{n(n-1)!}{2\pi}\left(\frac{2}{x_e}\right)^{n+1}, \tag{13}$$

the real and imaginary parts of the denominator are given by

$$\begin{aligned} \Re(D) = &\frac{1}{2n!}\left[nx_iJ_n(x_i) - x_e^2J_n'(x_i)\right]\left(\frac{x_e}{2}\right)^{n-1} \\ &+ \frac{1}{2(n+1)!}\left[-(n+2)x_iJ_n(x_i) + x_e^2J_n'(x_i)\right]\left(\frac{x_e}{2}\right)^{n+1}, \end{aligned} \tag{14}$$

$$\Im(D) = \frac{(n-1)!}{2\pi}\left[nx_iJ_n(x_i) - x_e^2J_n'(x_i)\right]\left(\frac{2}{x_e}\right)^{n+1}. \tag{15}$$

Now we consider the numerator of the Mie coefficient $a_n$,

$$N(x_e) = x_iJ_n(x_i)J_n'(x_e) - x_eJ_n(x_e)J_n'(x_i), \tag{16}$$

and expand the Bessel functions up to two terms,

$$J_n(x_e) \approx \frac{1}{n!}\left(\frac{x_e}{2}\right)^n - \frac{1}{(n+1)!}\left(\frac{x_e}{2}\right)^{n+2}, \tag{17}$$

$$J_n'(x_e) \approx \frac{1}{2(n-1)!}\left(\frac{x_e}{2}\right)^{n-1} - \frac{1}{2(n+1)!}\left(\frac{x_e}{2}\right)^{n+1}, \tag{18}$$

to get

$$\begin{aligned} N \approx &\left[nx_iJ_n(x_i) - x_e^2J_n'(x_i)\right]\left(\frac{x_e}{2}\right)^{n-1} \\ &- \frac{1}{2(n+1)!}\left[x_iJ_n(x_i) - x_e^2J_n'(x_i)\right]\left(\frac{x_e}{2}\right)^{n+1}. \end{aligned} \tag{19}$$

We see from (15) that the resonance condition is met by

$$n x_i J_n(x_i) - x_e^2 J_n'(x_i) = 0, \tag{20}$$

which for $x_e \to 0$ reduces to

$$J_n(x_i) = 0. \tag{21}$$

For the resonance width, we have

$$\gamma_n \propto \frac{-1}{2(n+1)!}\left[(n+2)x_i J_n(x_i) - x_e^2 J_n'(x_i)\right]\left(\frac{x_e}{2}\right)^{n+1}, \tag{22}$$

or

$$\gamma_n \propto \frac{1}{2(n+1)!}\left[x_e^2 J_n'(x_i)\right]\left(\frac{x_e}{2}\right)^{n+1}, \tag{23}$$

which tends to zero as $x_e \to 0$.

In the case where the electric field is polarized parallel to the cylinder axis (TM polarization), the extinction efficiency is again proportional to the real part of the scattering coefficients $b_n$, expressed as

$$b_n = \frac{x_e J_n(x_i) J_n'(x_e) - x_i J_n(x_e) J_n'(x_i)}{x_e J_n(x_i) H_n^{(1)\prime}(x_e) - x_i H_n^{(1)}(x_e) J_n'(x_i)}. \tag{24}$$

Using the small-argument expansions for $n = 0$,

$$H_0^{(1)}(x_e) \approx \left(1 - \frac{x_e^2}{4}\right) + i \cdot \frac{2}{\pi}\left(\ln\left(\frac{x_e}{2}\right) + \gamma\right), \tag{25}$$

$$H_0^{(1)\prime}(x_e) \approx -\frac{x_e}{2} + i \cdot \left(-\frac{2}{\pi x_e}\right), \tag{26}$$

$$J_0(x_e) \approx 1 - \frac{x_e^2}{4}, \tag{27}$$

$$J_0'(x_e) \approx -\frac{x_e}{2}, \tag{28}$$

the real and imaginary parts of the denominator become

$$\Re(D) = -\frac{x_e^2}{2} J_0(x_i) - x_i J_0'(x_i) \left(1 - \frac{x_e^2}{4}\right), \tag{29}$$

$$\Im(D) = -\frac{2}{\pi} J_0(x_i) - \frac{2x_i}{\pi} J_0'(x_i) \left(\ln\left(\frac{x_e}{2}\right) + \gamma\right), \tag{30}$$

and the numerator becomes

$$N \approx -\frac{1}{2} x_e^2 J_0(x_i) - x_i J_0'(x_i) \left(1 - \frac{x_e^2}{4}\right). \tag{31}$$

From these expressions we see that to remove the divergence of the denominator, we should have

$$J_0'(x_i) = 0, \tag{32}$$

and consequently, $\Re(D)$ vanishes when $x_e \to 0$. However, $\Im(D)$ remains non-vanishing in the small-size-parameter limit, preventing the emergence of a resonance condition. Thus, as can also be inferred from Fig. 2b of the main text, the $\mathrm{TM}_0$ mode of the infinite cylinder does not support a true resonance.

For $n > 0$ we use the small-argument approximations

$$H_n^{(1)}(x_e) \approx \frac{1}{n!} \left(\frac{x_e}{2}\right)^n + i \cdot \frac{(n-1)!}{\pi} \left(\frac{2}{x_e}\right)^n, \tag{33}$$

$$H_n^{(1)\prime}(x_e) \approx \frac{n}{2n!} \left(\frac{x_e}{2}\right)^{n-1} - i \cdot \frac{n(n-1)!}{2\pi} \left(\frac{2}{x_e}\right)^{n+1}, \tag{34}$$

to obtain the real and imaginary parts of the denominator as

$$\Re(D_n) = \left(\frac{x_e}{2}\right)^n \cdot \frac{1}{n!}\left[\frac{n}{2}J_n(x_i) - x_i J_n'(x_i)\right] + \left(\frac{x_e}{2}\right)^{n+2}\left[\frac{x_i J_n'(x_i)}{(n+1)!} - \frac{(n+2)}{2(n+1)!}J_n(x_i)\right], \quad (35)$$

$$\Im(D_n) = -\left(\frac{2}{x_e}\right)^n \cdot \frac{(n-1)!}{\pi}\left[\frac{n}{2}J_n(x_i) + x_i J_n'(x_i)\right]. \quad (36)$$

Using only the leading-order terms of the Bessel functions,

$$J_n(x_e) \approx \frac{1}{n!}\left(\frac{x_e}{2}\right)^n, \quad J_n'(x_e) \approx \frac{1}{2(n-1)!}\left(\frac{x_e}{2}\right)^{n-1}, \quad (37)$$

the numerator becomes

$$N \approx \left(\frac{x_e}{2}\right)^n \cdot \frac{1}{n!}\left[\frac{n}{2}J_n(x_i) - x_i J_n'(x_i)\right]. \quad (38)$$

Hence the resonance condition is satisfied when

$$\frac{n}{2}J_n(x_i) + x_i J_n'(x_i) = 0, \quad (39)$$

and for the resonance width we have

$$\gamma_n \propto \Re(D_n), \quad (40)$$

indicating that the resonance width tends to zero as $x_e \to 0$.

## Section B: Mie Resonances of Spherical Particles in ENZ Media

To analyze the ENZ limit $x_e \to 0$, we use the small-argument expansions of the functions

$$\psi_n(x_e) = \frac{x_e^{n+1}}{(2n+1)!!} + \mathcal{O}(x_e^{n+3}), \quad \psi_n'(x_e) = \frac{(n+1)x_e^n}{(2n+1)!!} + \mathcal{O}(x_e^{n+2}), \quad (41)$$

$$\xi_n(x_e) = \frac{x_e^{n+1}}{(2n+1)!!} + i\left(-\frac{(2n-1)!!}{x_e^n}\right) + \mathcal{O}(x_e^{n+3}) + i\mathcal{O}(x_e^{2-n}), \tag{42}$$

$$\xi_n'(x_e) = \frac{(n+1)x_e^n}{(2n+1)!!} + i\left(n\frac{(2n-1)!!}{x_e^{n+1}}\right) + \mathcal{O}(x_e^{n+2}) + i\mathcal{O}(x_e^{1-n}), \tag{43}$$

and write the real part of $b_n$ in terms of its numerator ($N$) and denominator ($D$) as

$$\Re(b_n) \approx \Re\left(\frac{1}{D}\right) N = \frac{\Re(D)N}{\Re(D)^2 + \Im(D)^2}. \tag{44}$$

Then the numerator $N$, $\Re(D)$ and $\Im(D)$ are calculated as

$$N = \frac{x_e^{n+1}}{(2n+1)!!}\left((n+1)\psi_n(x_i) - x_i\psi_n'(x_i)\right) + \mathcal{O}(x_e^{n+3}), \tag{45}$$

$$\Re(D) = \frac{x_e^{n+1}}{(2n+1)!!}\left((n+1)\psi_n(x_i) - x_i\psi_n'(x_i)\right) + \mathcal{O}(x_e^{n+3}), \tag{46}$$

$$\Im(D) = \frac{(2n-1)!!}{x_e^n}\left(n\psi_n(x_i) + x_i\psi_n'(x_i)\right) + \mathcal{O}(x_e^{2-n}). \tag{47}$$

From these results we see that the dipolar or multipolar magnetic resonances occur when $\Im(D) = 0$ or

$$n\psi_n(x_i) + x_i\psi_n'(x_i) = 0, \tag{48}$$

which is consistent with the blue curves in Fig. 3b of the main text. For the resonance widths we get

$$\gamma_n \propto \frac{x_e^{n+1}}{(2n+1)!!}\left((n+1)\psi_n(x_i) - x_i\psi_n'(x_i)\right), \tag{49}$$

tending to zero as $x_e \to 0$.

Similarly, for the $a_n$ coefficients we obtain

$$N = \frac{x_e^n}{(2n+1)!!}\left((n+1)x_i\psi_n(x_i) - x_e^2\psi_n'(x_i)\right) + \mathcal{O}(x_e^{n+2}), \tag{50}$$

$$\Re(D) = \frac{x_e^n}{(2n+1)!!}\left((n+1)x_i\psi_n(x_i) - x_e^2\psi_n'(x_i)\right) + \mathcal{O}(x_e^{n+2}), \tag{51}$$

$$\Im(D) = (2n-1)!! \left( \psi_n(x_i) n \frac{x_i}{x_e^{n+1}} + \psi_n'(x_i) \right) + \mathcal{O}(x_e^{1-n}), \tag{52}$$

indicating that in the ENZ regime ($x_e \to 0$), the resonance width, proportional to $\Re(D)$, approaches zero. Although the divergence of the first term in $\Im(D)$ suggests that resonances are suppressed in this regime, a more careful analysis reveals that for any arbitrarily small $x_e$, there always exist roots of $\Im(D)$ given by

$$x_i = \frac{\alpha}{2} + \frac{1}{2n}\sqrt{n^2\alpha^2 - 4n x_e^{n+1}}, \tag{53}$$

located near the zeros $\alpha$ of the function $\psi_n(x)$, which is consistent with the red curves in Fig. 3b.

## The roots of $\Im(D)$

Let $\alpha$ denote a simple zero of $\psi_n(x_i)$, that is,

$$\psi_n(\alpha) = 0, \quad \text{and} \quad \psi_n'(\alpha) \neq 0. \tag{54}$$

Expanding $\psi_n(x_i)$ near $\alpha$ gives

$$\psi_n(x_i) \approx \psi_n'(\alpha)(x_i - \alpha), \tag{55}$$

and also

$$\psi_n'(x_i) \approx \psi_n'(\alpha). \tag{56}$$

Substituting into the function

$$f(x_i) = \psi_n(x_i) n \frac{x_i}{x_e^{n+1}} + \psi_n'(x_i), \tag{57}$$

we find

$$f(x_i) \approx \psi_n'(\alpha)\left(n\frac{x_i(x_i-\alpha)}{x_e^{n+1}}+1\right). \tag{58}$$

Setting $f(x_i)=0$, we obtain

$$nx_i(x_i-\alpha)+x_e^{n+1}=0, \tag{59}$$

or equivalently,

$$nx_i^2-n\alpha x_i+x_e^{n+1}=0, \tag{60}$$

which has the solutions

$$x_i=\frac{\alpha}{2}\pm\frac{1}{2n}\sqrt{n^2\alpha^2-4nx_e^{n+1}}. \tag{61}$$

As $x_e\to 0$, the square root behaves like

$$\sqrt{n^2\alpha^2-4nx_e^{n+1}}\approx n\alpha, \tag{62}$$

thus the + root approaches $x_i=\alpha$ while the $-$ root approaches $x_i=0$. Since we seek a root near $\alpha$, we select the positive-sign root,

$$x_i=\frac{\alpha}{2}+\frac{1}{2n}\sqrt{n^2\alpha^2-4nx_e^{n+1}}. \tag{63}$$

## Section C: Electric and Magnetic Line Sources, Green Tensor, LDOS, and Purcell Factor

### C.1. Conventions

We use time-harmonic fields with the phasor convention $e^{-i\omega t}$. Free-space constants are $\varepsilon_0$ and $\mu_0$, with $c=1/\sqrt{\varepsilon_0\mu_0}$ and $k_0=\omega/c$. Throughout, material permeabilities are taken as $\mu(\mathbf{r},\omega)=\mu_0$.

## C.2. Electric dipole: field and Purcell factor

Applying the inhomogeneous vector Helmholtz equation

$$\nabla \times \nabla \times \mathbf{E}(\mathbf{r}) - k_0^2\, \varepsilon(\mathbf{r},\omega)\, \mathbf{E}(\mathbf{r}) = i\omega\mu_0\, \mathbf{J}(\mathbf{r}), \tag{64}$$

the electric dyadic Green tensor $\mathbf{G}(\mathbf{r},\mathbf{r}';\omega)$ is defined as the solution of

$$\nabla \times \nabla \times \mathbf{G}(\mathbf{r},\mathbf{r}';\omega) - k_0^2\, \varepsilon(\mathbf{r},\omega)\, \mathbf{G}(\mathbf{r},\mathbf{r}';\omega) = \mathbf{I}\, \delta(\mathbf{r}-\mathbf{r}'), \tag{65}$$

satisfying the outgoing-wave radiation condition. The total field produced by an arbitrary current distribution $\mathbf{J}(\mathbf{r}')$ is obtained as

$$\mathbf{E}(\mathbf{r}) = i\omega\mu_0 \int \mathbf{G}(\mathbf{r},\mathbf{r}';\omega)\, \mathbf{J}(\mathbf{r}')\, \mathrm{d}^2 r'. \tag{66}$$

In a two-dimensional formulation the elementary emitter is an electric line current directed along the $z$ axis:

$$\mathbf{J}(\mathbf{r}) = \hat{\mathbf{z}}\, J_0\, \delta(\mathbf{r}-\mathbf{r}_0). \tag{67}$$

Substitution into the Green representation yields

$$\mathbf{E}(\mathbf{r}) = i\omega\mu_0 \mathbf{G}(\mathbf{r},\mathbf{r}_0)\mathbf{J}_0, \qquad \mathbf{E}(\mathbf{r}_0) = i\omega\mu_0 \mathbf{G}(\mathbf{r}_0,\mathbf{r}_0)\mathbf{J}_0. \tag{68}$$

The power emitted by a harmonic current source is

$$P = \frac{1}{2}\Re\left\{\mathbf{J}^* \cdot \mathbf{E}(\mathbf{r}_0)\right\}. \tag{69}$$

Substituting the Green tensor expression gives

$$P \propto \mathbf{J}^* \cdot \Im\left[\mathbf{G}(\mathbf{r}_0, \mathbf{r}_0)\right] \cdot \mathbf{J}. \tag{70}$$

Thus the emitted power is proportional to the imaginary part of the dyadic Green tensor at the emitter position, which corresponds to the local density of electromagnetic states.

The Purcell factor is defined as the ratio of emitted power in the structure and in the homogeneous background medium:

$$F_P(\omega) = \frac{P}{P_0}. \tag{71}$$

For an out-of-plane electric line source

$$\mathbf{J}(\mathbf{r}) = \hat{\mathbf{z}} J_0 \delta(\mathbf{r} - \mathbf{r}_0),$$

the emitted power becomes

$$P = \frac{1}{2}\Re\{J_0^* E_z(\mathbf{r}_0)\}. \tag{72}$$

Similarly, in the homogeneous background medium

$$P_0 = \frac{1}{2}\Re\{J_0^* E_{z,0}(\mathbf{r}_0)\}. \tag{73}$$

Since the same source amplitude $J_0$ is used in both calculations, it cancels in the ratio, giving

$$F_P(\omega) = \frac{\Re\{E_z(\mathbf{r}_0, \omega)\}}{\Re\{E_{z,0}(\mathbf{r}_0, \omega)\}}. \tag{74}$$

Thus the Purcell factor can be obtained directly from the electric field at the source position normalized by the field produced by the same line source in the homogeneous background.

## C.3. Magnetic line source: field and Purcell factor

Magnetic emitters can be treated by introducing a fictitious magnetic current density

$$\nabla \times \mathbf{E} = i\omega\mu_0\mathbf{H} - \mathbf{M}, \tag{75}$$

$$\nabla \times \mathbf{H} = -i\omega\varepsilon_0\varepsilon(\mathbf{r},\omega)\mathbf{E}. \tag{76}$$

For a magnetic line source oriented along $z$

$$\mathbf{M}(\mathbf{r}) = \hat{\mathbf{z}}M_0\delta(\mathbf{r}-\mathbf{r}_0), \tag{77}$$

the emitted power is

$$P_m = \frac{1}{2}\Re\{\mathbf{M}^* \cdot \mathbf{H}(\mathbf{r}_0)\} = \frac{1}{2}\Re\{M_0^* H_z(\mathbf{r}_0)\}\,. \tag{78}$$

In the homogeneous background medium

$$P_{m,0} = \frac{1}{2}\Re\{M_0^* H_{z,0}(\mathbf{r}_0)\}\,. \tag{79}$$

Therefore the magnetic Purcell factor becomes

$$F_{P,m}(\omega) = \frac{\Re\{H_z(\mathbf{r}_0,\omega)\}}{\Re\{H_{z,0}(\mathbf{r}_0,\omega)\}}. \tag{80}$$

Hence the magnetic Purcell factor can also be determined by comparing the magnetic field at the source location in the structure with the corresponding field in the homogeneous background medium.

## Section D: The role of ENZ Condition on PF Enhancement

To clarify the role of the ENZ condition in the enhancement of the magnetic PF reported in Fig. 13, we investigated whether comparable enhancement can be achieved outside the ENZ regime. Two approaches were considered to move the system away from ENZ operation:

### 1. Reducing the lateral size of the cavity

In the ENZ regime, the effective wavelength scales inversely with the effective refractive index, $\lambda_{\text{eff}} = \lambda/n_{\text{eff}}$, and is therefore substantially larger than the free-space wavelength $\lambda$. In this regime, the lateral cavity length associated with the fundamental ENZ mode is approximately $L \approx \lambda/(2n_{\text{eff}})$,[1] indicating that ENZ operation requires cavity dimensions much larger than the free-space wavelength.

As shown in Fig. S1, reducing the cavity size from 18.75 $\mu$m in Fig. 13 to 12.75 $\mu$m and 6.75 $\mu$m decreases the PF from 833 to 400 and 108, respectively. This behavior reflects the progressive deviation from the ENZ condition as the cavity size is reduced. Consequently, the suppression of radiative losses in the NC Mie resonators becomes less effective, leading to a significant reduction in both the $Q$-factor and the PF.

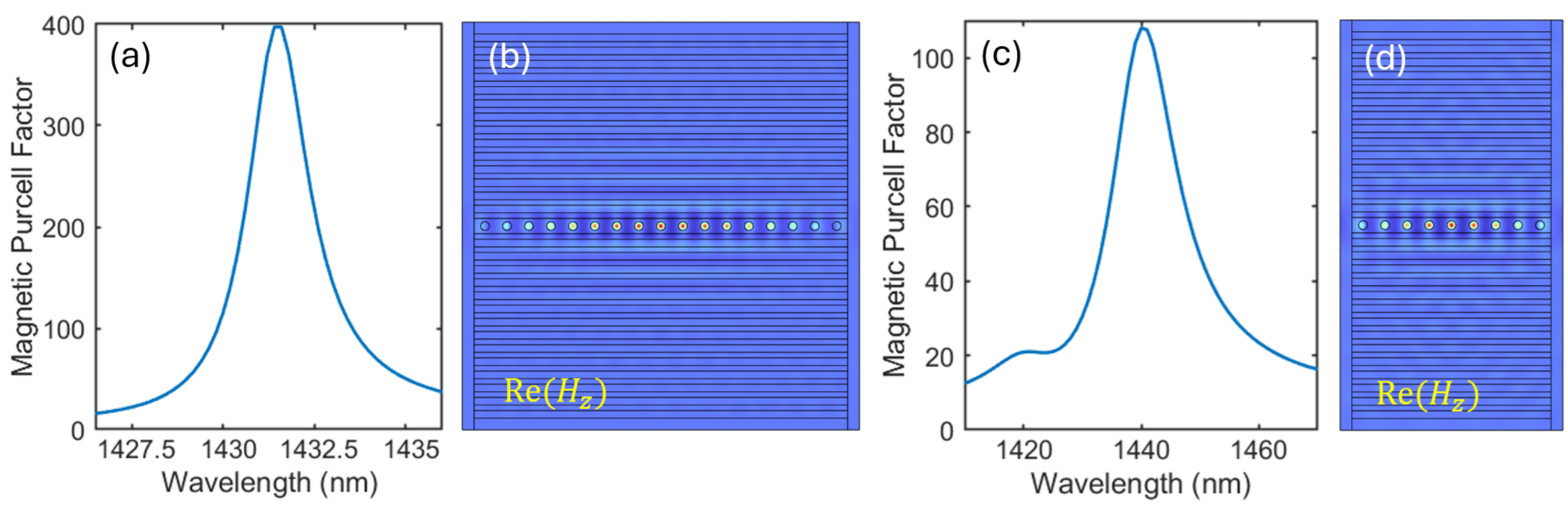


Figure S1: (a,c) Magnetic PF spectra and (b,d) corresponding Spatial magnetic field distribution, Re($H_z$), for reduced cavity sizes. The cavity length is decreased from 18.75 $\mu$m (Fig. 13) to 12.75 $\mu$m in (a,b) and 6.75 $\mu$m in (c,d).

### 2. Shifting the cutoff of the undoped structure away from the NC Mie resonance by increasing the core thickness

Figure S2a shows the transmission spectrum of a Bragg cavity with a cutoff wavelength of 1500 nm. The spectral region between the near-cutoff resonance ($\sim$ 1500 nm) and the lower edge of the stopband ($\sim$ 1350 nm) spans approximately 150 nm. As shown in Fig. S2b, the effective index within this region remains below unity and approaches zero near the cutoff, defining the ENZ regime of the structure.

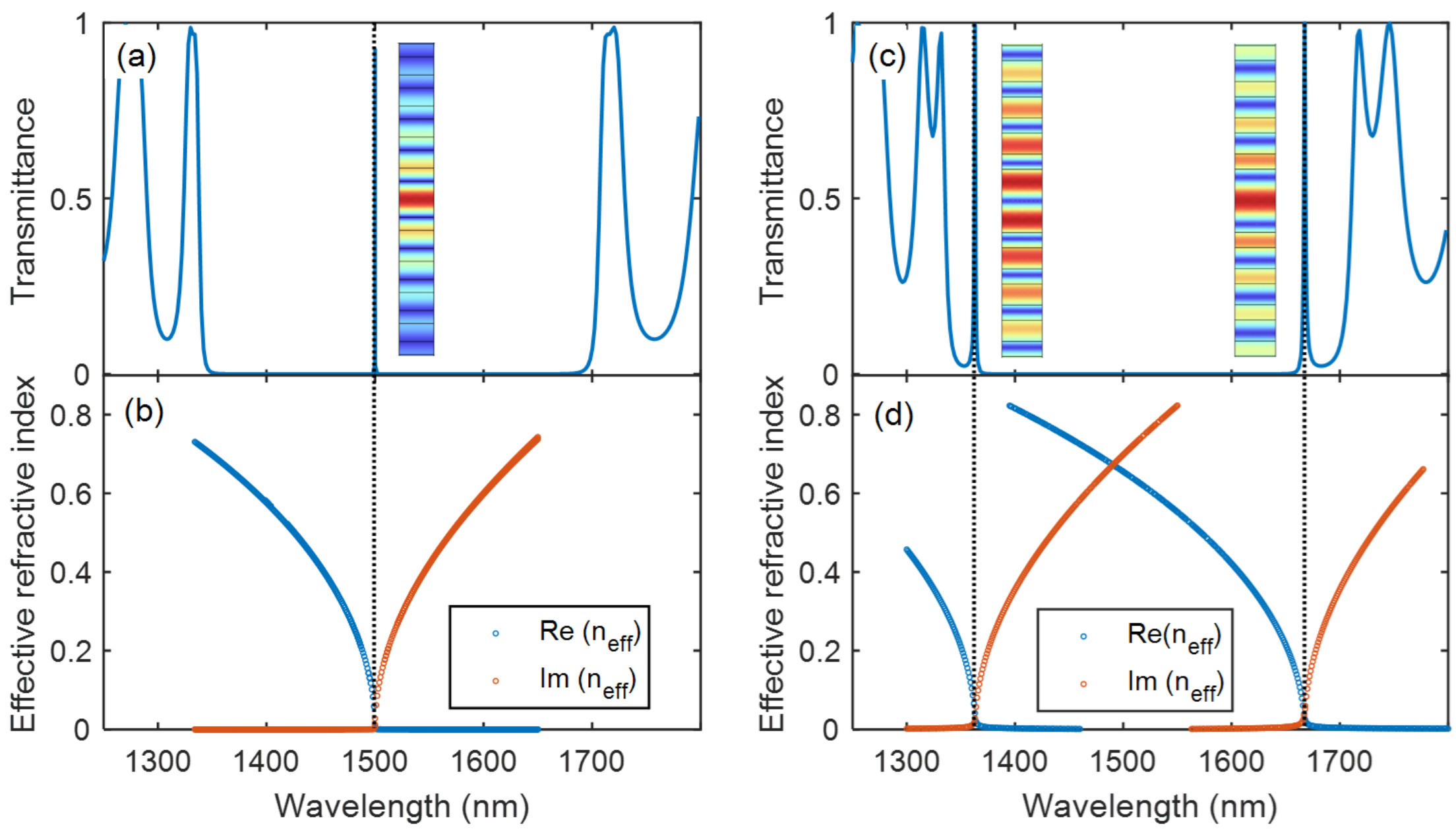


Figure S2: Transmittance spectra and the corresponding electric-field profile and effective refractive index of Bragg cavities. (a,b) Structure with cutoff $\lambda_c \approx 1500$ nm and core thickness 520.83 nm, showing an ENZ region near the cutoff. (c,d) Increased core thickness to 781.25 nm shifts the resonant mode to $\lambda_c \approx 1667.5$ nm and introduces a higher-order mode at $\lambda \approx 1363.1$ nm with its own ENZ region.

The magnetic Mie resonance of the NC in free space (Fig. 1 of the manuscript) exhibits a broad spectral bandwidth of approximately 300 nm, which is larger than the ENZ spectral window and comparable to the whole stopband width. This ensures substantial spectral overlap between the Mie resonance and the ENZ region, enabling Bragg-Mie modal hybridization, even if the cutoff is shifted toward the higher edge of the stopband. For example, when the core thickness is increased from 520.83 nm (in Fig. S2(a,b)) to 781.25 nm, the resonant mode shifts to the longer wavelength of 1667.5 nm, as shown in Fig. S2c. However, as seen in Fig. S2d, the effective index is still less than unity in a wide range within the

stopband and even a higher-order mode emerges at $\lambda = 1363.1$ nm, possessing its own cutoff point and corresponding ENZ region (Fig. S2d), which can lead to hybridization of the NC Mie resonance with the higher order ENZ mode.

Further increase in core thickness results in emergence of even higher-order modes (Fig. S3) with their corresponding cutoffs and ENZ regions. As a result, increasing the core thickness does not ensure elimination of the spectral overlap between the Mie resonance and ENZ regions, and even introduces multiple modal branches capable of supporting ENZ behavior.

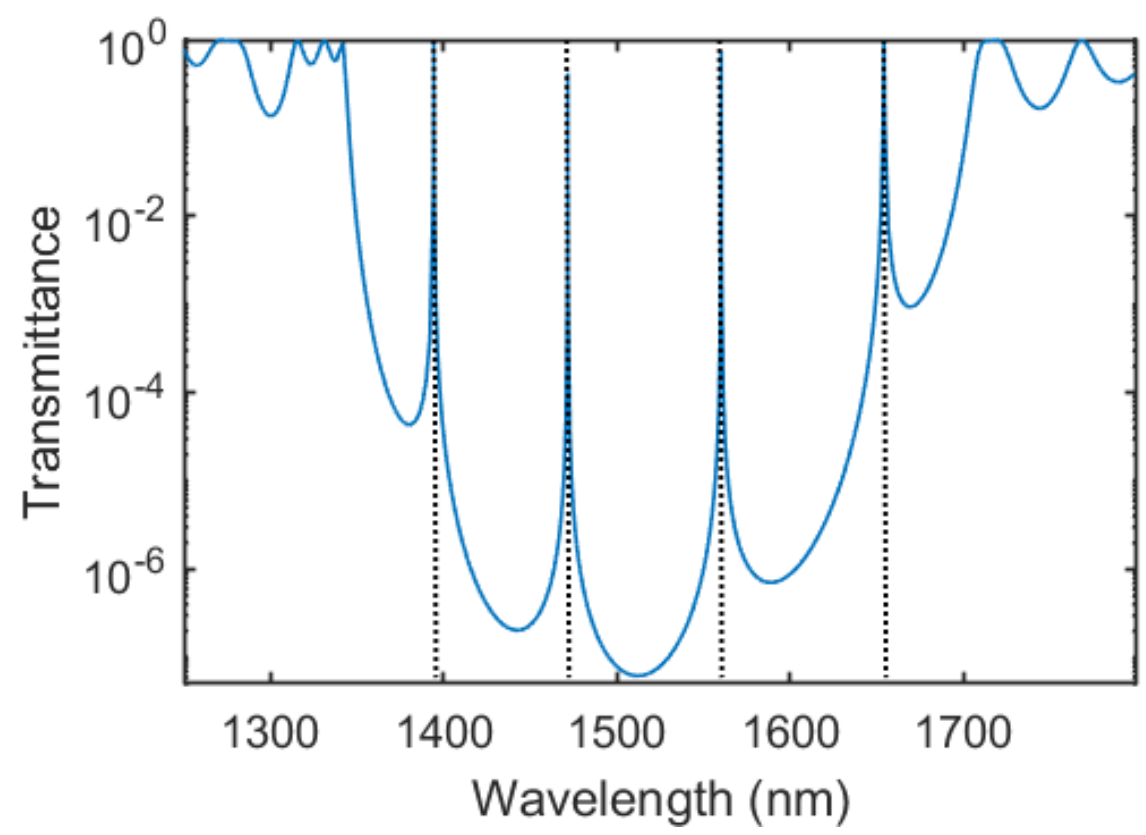


Figure S3: Transmittance spectrum of a Bragg cavity with the core thickness of 20 $\mu$m , showing the emergence of multiple higher-order modes within the mirrors' stopband. Each mode exhibits a distinct cutoff wavelength (indicated by dashed lines) and an associated ENZ region.

As discussed in Sec. 3.4 and Fig. 14, a slight increase in the core thickness with respect to its value in Fig. 13, can even lead to optimized PF values as high as $1.8 \times 10^4$. However, increasing the core thickness beyond its optimal value reduces the magnetic PF. For example, Fig. S4 presents the magnetic PF and corresponding field distributions for the same structure as in Fig. 14, but with a core thickness of 20 $\mu$m.

In this regime, although the cavity $Q$ continues to increase with increasing core thickness, the spatial overlap between the bare-cavity ENZ mode and the NC Mie mode progressively diminishes. As illustrated in Fig. S4b and S4c, the hybrid mode becomes increasingly delocalized across the enlarged core region between the Bragg mirrors, resulting in a substantially

larger effective mode volume compared to the optimized case (Fig. 14), where the field is predominantly confined within or in the immediate vicinity of the NCs.

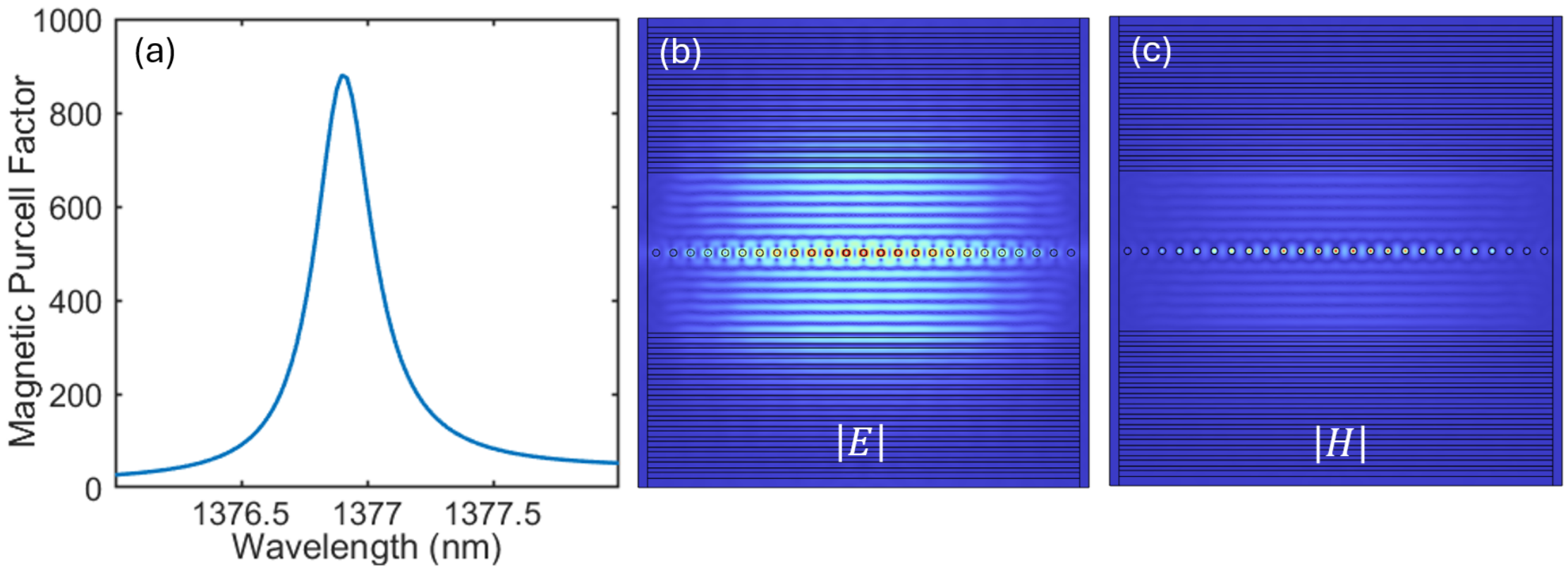


Figure S4: (a) Magnetic PF and (b,c) field distributions for the same structure as in Fig. 14, but with an increased core thickness of 20 $\mu$m.

While, in principle, increasing the core thickness does not preclude realization of the ENZ condition, in practice the realization of bulk slab cores with sufficiently high surface parallelism and flatness is challenging. Even slight curvature or non-parallelism can perturb the near-zero in-plane wavevector condition ($k_{\|} \approx 0$) required to sustain the ENZ regime. This constraint is significantly relaxed in Bragg cavities with thin-film (half-wave) cores, where the use of high-flatness substrates and standard deposition techniques enables the realization of highly parallel interfaces with minimal thickness nonuniformity, thereby preserving the ENZ condition with enhanced robustness.

## Section E: Electric Purcell Enhancement in Doped ENZ Bragg Cavities Excited by TM-Polarized Waves

The electric Purcell enhancement in NC-doped Bragg cavities can be investigated for the fundamental as well as higher order NZI TM-polarized modes by exciting the structure with electric line currents placed at the electric hot spots of the modes. Figure S5 presents the corresponding calculations for the same cavity configuration as in Fig. 13 in the article, but with lattice period of $p = 500\,\text{nm}$ and a cavity size of $W = 12.5\,\mu\text{m}$ and with electric rather

than magnetic line-current excitation. The resulting electric PF spectrum, shown in Fig. S5a, exhibits a series of sharp resonances corresponding to different orders of NZI TM-like modes. The electric and magnetic field distributions for the lowest-order mode at $\lambda = 1399.8$ nm are shown in Figs. S5b and S5c, respectively. Both profiles demonstrate strong field localization within the NCs, with the inset zoom-ups highlighting the distinct electric and magnetic hot spots of the resonant mode.

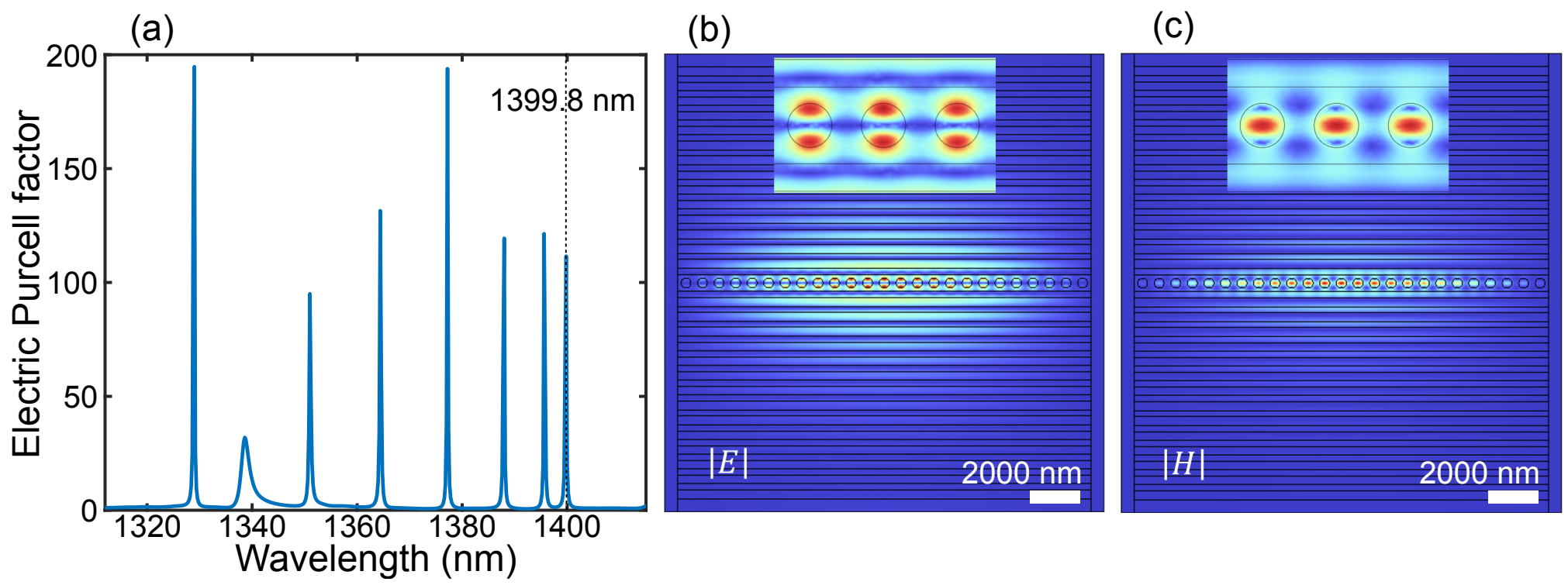


Figure S5: Electric Purcell enhancement in a Bragg cavity with $N = 14$ cladding-layer pairs and width $W = 12.5$ $\mu$m, loaded with an array of 25 silicon NCs of radius $R = 150$ nm and period $p = 500$ nm. (a) Electric Purcell factor spectrum (b) Electric field distribution $|E|$ and (c) magnetic field distribution $|H|$ for the lowest-order TM-like mode at $\lambda = 1399.8$ nm.

## Section F: Impact of Optical Losses on PD Performance

To evaluate the impact of optical losses on device performance, we computed the $Q$ factor and PF while increasing material losses in the structure shown in Fig. 14. Specifically, the imaginary part of the refractive index (representing optical losses) for $SiO_2$, SiN, and Si was increased from their bulk values of $\kappa_{SiO_2} = 10^{-6}$, $\kappa_{SiN} = 10^{-7}$, and $\kappa_{Si} = 4 \times 10^{-13}$ to $\kappa_{SiO_2} = \kappa_{SiN} = \kappa_{Si} = 10^{-5}$ and $10^{-4}$, corresponding to Figs. S6a and S6b, respectively. As shown in these figures, despite this substantial increase in material losses, the structure maintains strong performance, with $Q = 2.56 \times 10^4$ and PF $= 1.4 \times 10^4$ for Fig. S6a, and $Q = 7.25 \times 10^3$ and PF $= 4.4 \times 10^3$ for Fig. S6b. These results indicate that the proposed structure remains robust against significant optical losses.

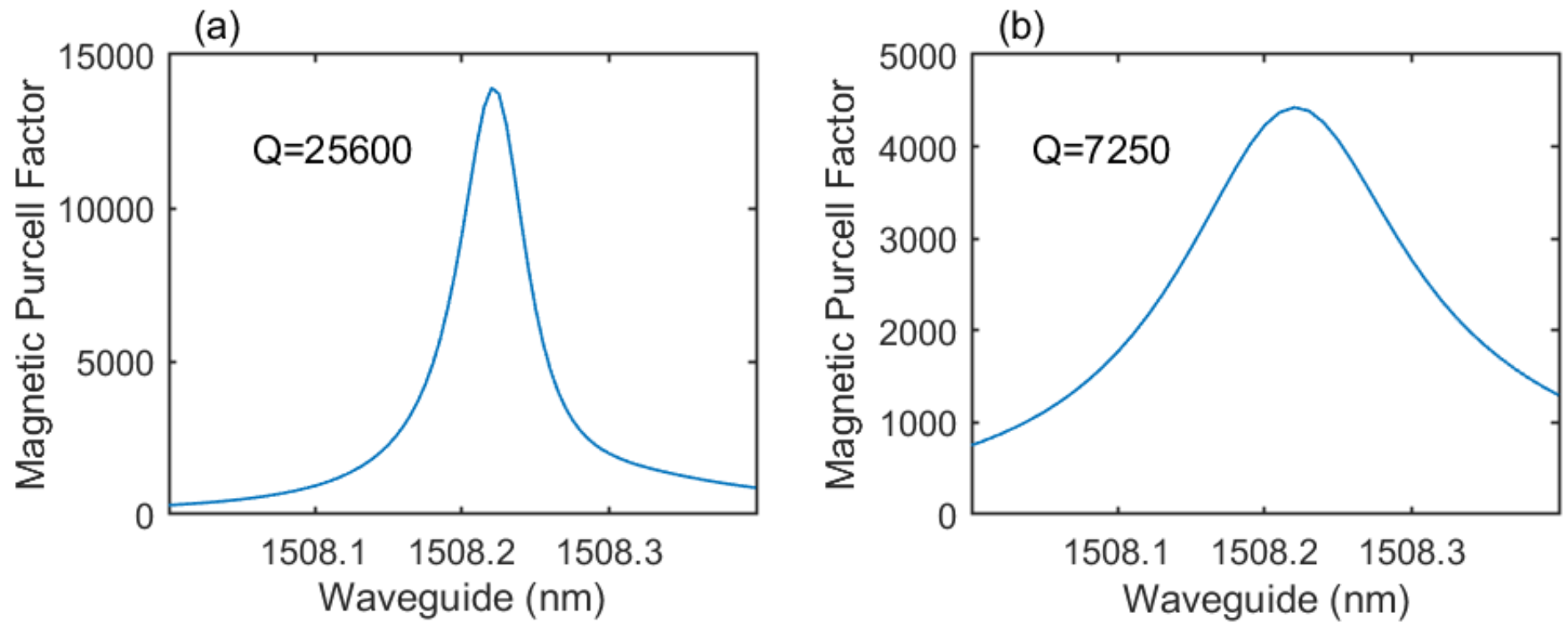


Figure S6: Computed PF of the same structure in Fig. 14 but with increased material losses.

We note that fabrication imperfections in nanophotonic structures, such as surface roughness, interface disorder, and dimensional variations, primarily introduce additional scattering and absorption channels, which to first order can be effectively modeled as increased material losses (i.e., higher $\kappa$). Therefore, this approach provides a rough estimate of fabrication-induced degradation, and the results demonstrate the robustness of the structure against such imperfections.

## Section G: Feasibility of Fabrication

We note that although NCs with circular cross sections are considered as dopants in this work—owing to the availability of closed-form analytical Mie coefficients—the photonic doping concept is not restricted to this geometry. In practice, inclusions with rectangular or square cross sections can also be employed, which are significantly easier to fabricate. Fig. S7(a) shows that a high $Q$-factor ($Q = 1.84 \times 10^4$) and significant Purcell enhancement (PF $= 5.82 \times 10^3$) can also be achieved at the center of a doped Bragg cavity using NC inclusions with square, rather than circular, cross sections. All other structural parameters and excitation conditions are identical to those in Fig. 14. Figures S7b and S7c show the corresponding electric and magnetic field distributions at the resonance wavelength $\lambda = 1572.21$ nm, exhibiting strong field confinement in the central region of the cavity.

Furthermore, similar Bragg structures incorporating embedded subwavelength metasur-

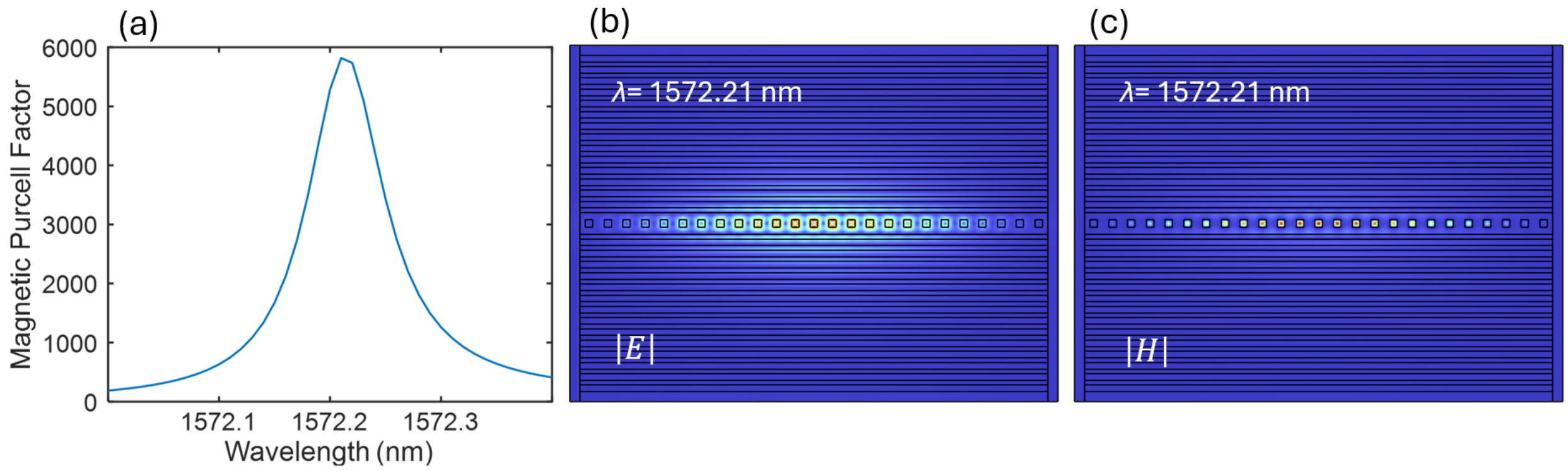


Figure S7: (a) Magnetic PF spectrum for the same structure and excitation conditions as in Fig. 14, with circular-cross-section NC dopants replaced by square-cross-section inclusions. (b) Electric and (c) magnetic field distributions inside the doped cavity.

faces (e.g., one- or two-dimensional gating structures) have already been experimentally demonstrated, particularly in polymer-based cavity cores, for tunable optical filtering applications.[2–5] In these implementations, the Bragg mirrors are formed by low-loss dielectric multilayers (e.g., Si/$SiO_2$), while the polymer is used as the core material to embed and planarize the metasurface layer. Typically, bottom Bragg reflector (BR) stacks are first deposited on a substrate, followed by patterning of subwavelength metasurface elements (e.g., Si nano-posts or gratings) using electron-beam lithography and dry etching. The metasurface is then embedded and planarized using a spin-coated polymer layer (e.g., SU-8), which forms the core medium, after which the top BR is deposited to complete the Fabry-Perot structure. Here, two alternative fabrication approaches are also presented that avoid polymer materials and thereby reducing optical losses and improving performance:

### Fabrication Method I: Damascene Embedding of Silicon Inclusions

A damascene-style embedding process enables precise positioning of silicon inclusions within a planar cavity defect layer. The bottom BR is first deposited using alternating layers of silicon dioxide ($SiO_2$) and a high-index dielectric (SiN or $Ta_2O_5$) by PECVD or, where reduced optical loss is required, LPCVD or IBS. A partial $SiO_2$ defect layer is then deposited.

The inclusion geometry is defined using deep-UV or electron-beam lithography and trans-

ferred into the defect layer by anisotropic RIE (or ICP-RIE), forming recessed cavities with controlled sidewall profiles. Silicon, typically amorphous or polycrystalline, is deposited conformally to fill the etched regions. Chemical mechanical polishing (CMP) removes excess silicon and restores surface planarity, leaving silicon confined within the predefined recesses. The defect layer is then completed, and the top BR is deposited symmetrically.

### Fabrication Method II: Crystalline Silicon Transfer via SOI Bonding

For applications requiring reduced absorption and improved optical performance, crystalline silicon inclusions can be realized via silicon-on-insulator (SOI) layer transfer. The SOI device layer is patterned lithographically and etched to define the inclusions. The structured SOI wafer is then bonded to the partially fabricated cavity substrate using plasma-assisted oxide bonding.

The handle silicon is removed by mechanical thinning followed by selective wet etching to the buried oxide (BOX), which is subsequently eliminated using HF-based etching. This process leaves crystalline silicon inclusions embedded at the defect plane. The cavity is completed by depositing the remaining dielectric layers and the top BR stack.

Compared to deposited amorphous or polycrystalline silicon, this approach provides superior material quality and reduced absorption. For mirrors targeting even lower loss and higher reflectivity, $Ta_2O_5$ in the BR stack may be replaced by SiN, which offers reduced absorption[6] in the telecom band and improved compatibility with high-Q cavity implementations, with a moderate reduction in refractive index contrast.

## Sec. H: Experimental Characterization and Measurement Methods

The cavity $Q$ factor can be extracted from spectral measurements of transmission or reflection under near-normal illumination of the Bragg-cavity structure. The Purcell enhancement can be evaluated through standard spontaneous emission rate measurements or time-resolved photoluminescence (lifetime) measurements using embedded emitters.

As illustrated in Fig. S8a, the embedded emitters can be optically excited by a pump wave with an appropriate wavelength and angle of incidence matched to a targeted NZI resonant Bragg-Mie mode. The resulting emission can be detected at a wavelength and emission angle associated with other resonant modes. Selective excitation of specific modes can be achieved by controlling the polarization and tuning the angle of incidence, while monitoring the transmitted and/or reflected spectra.

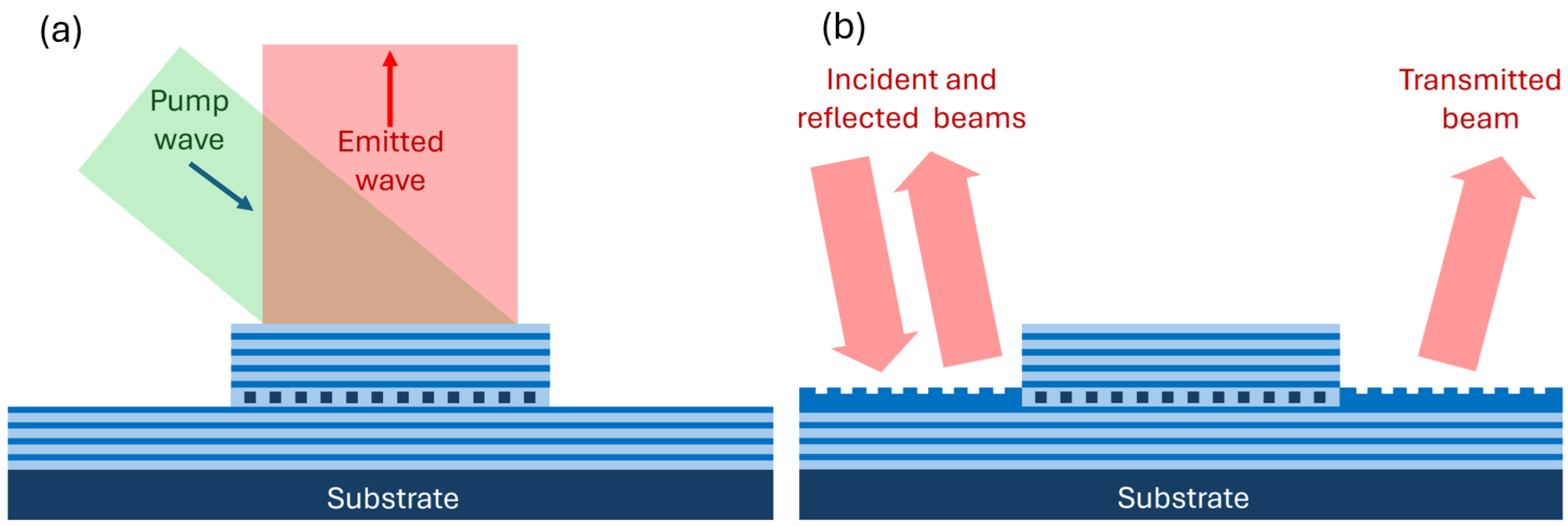


Figure S8: Experimental characterization schemes: (a) Purcell enhancement measurement using optically excited emitters coupled to NZI Bragg-Mie modes in the cavity. (b) Retrieval of effective electromagnetic parameters via input/output coupling gratings by measuring the reflected and transmitted beams.

Finally, the effective electromagnetic parameters of the structure, including the EMNZ response (effective refractive index, permittivity, and permeability), can be retrieved using standard parameter-extraction techniques based on reflection/transmission measurements. This can be implemented by incorporating input/output coupling gratings, as schematically shown in Fig. S8b, and measuring the reflectance and transmittance of the incident waves.

# References


(1) A. Panahpour, J. Kelavuori, and M. Huttunen, "Purcell Effect in Epsilon-Near-Zero Microcavities," *ACS Omega*, vol. 10, no. 38, pp. 44683–44692, 2025.

(2) Y. Horie, A. Arbabi, E. Arbabi, S. M. Kamali, and A. Faraon, "Wide bandwidth and

high resolution planar filter array based on DBR-metasurface-DBR structures," *Opt. Express*, vol. 24, no. 11, pp. 11677–11682, 2016.

(3) C. Zhou, O. J. Martin, and E. Charbon, "Planar 16-band metasurface-enhanced spectral filter for integrated image sensing," *Opt. Express*, vol. 32, no. 5, pp. 7463–7472, 2024.

(4) A. McClung, S. Samudrala, M. Torfeh, M. Mansouree, and A. Arbabi, "Snapshot spectral imaging with parallel metasystems," *Sci. Adv.*, vol. 6, no. 38, p. eabc7646, 2020.

(5) L. Xiong, Y. Zhang, X. Lu, G. Xiang, Z. Zhang, and Y. Yuan, "High-quality structural color filter based on embedded metasurface and dielectric-metal cavity," *Opt. Commun.*, p. 132259, 2025.

(6) G. Rempe, R. J. Thompson, H. J. Kimble, and R. Lalezari, "Measurement of ultralow losses in an optical interferometer," *Optics Letters*, vol. 17, no. 5, pp. 363–365, 1992.
